# Deep embeddings to comprehend and visualize microbiome protein space

**DOI:** 10.1038/s41598-022-14055-7

**Published:** 2022-06-20

**Authors:** Krzysztof Odrzywolek, Zuzanna Karwowska, Jan Majta, Aleksander Byrski, Kaja Milanowska-Zabel, Tomasz Kosciolek

**Affiliations:** 1 Ardigen, Podole 76, 30-394 Krakow, Poland; 2 Institute of Computer Science, Faculty of Computer Science, Electronics and Telecommunications, AGH University of Science and Technology, Mickiewicza 30, 30-059 Krakow, Poland; 3 Malopolska Centre of Biotechnology, Jagiellonian University, Gronostajowa 7A, 30-387 Krakow, Poland; 4 Department of Computational Biophysics and Bioinformatics, Faculty of Biochemistry, Biophysics and Biotechnology, Jagiellonian University, Gronostajowa 7, 30-387 Krakow, Poland

**Keywords:** Microbiome, Protein sequence analyses, Machine learning

## Abstract

Understanding the function of microbial proteins is essential to reveal the clinical potential of the microbiome. The application of high-throughput sequencing technologies allows for fast and increasingly cheaper acquisition of data from microbial communities. However, many of the inferred protein sequences are novel and not catalogued, hence the possibility of predicting their function through conventional homology-based approaches is limited, which indicates the need for further research on alignment-free methods. Here, we leverage a deep-learning-based representation of proteins to assess its utility in alignment-free analysis of microbial proteins. We trained a language model on the Unified Human Gastrointestinal Protein catalogue and validated the resulting protein representation on the bacterial part of the SwissProt database. Finally, we present a use case on proteins involved in SCFA metabolism. Results indicate that the deep learning model manages to accurately represent features related to protein structure and function, allowing for alignment-free protein analyses. Technologies that contextualize metagenomic data are a promising direction to deeply understand the microbiome.

## Introduction

In just over a decade, a substantial body of evidence linked gut microbiome dysbiosis with diseases ranging from obesity^1^, inflammatory bowel disease^2–4^, diabetes^5,6^, cancer^7,8^, depression^9^ and other psychiatric disorders^10,11^. It shows the profound impact of the microbiome on human health and is a testament to rapid technological progress in sequencing technologies. Since the mid-2000s, the bulk of our insight into the role of the microbiome came from high-throughput and cost-effective 16S rRNA marker gene sequencing experiments that allow for taxonomic discrimination between microorganisms. Though informative, microbiome analysis based solely on taxonomy is prone to bias, due to incomplete reference databases and does not provide detailed information about microbiome function^12^. One of the areas of high interest and relevance is our ability to deduce the gene function from sequence, as it provides more insight into the microbiome’s role in human health. Functional analysis of microbiome data can be performed based on high-throughput, large-scale shotgun metagenomics and other multi-omics experiments that are now becoming accessible for large-scale studies. Gene sequence fragments generated during a shotgun sequencing experiment can be functionally annotated, using homology-based tools such as BLAST^13^ or HMMER^14^ that search fragments of sequences against reference databases such as Pfam or Gene Ontology (GO)^15^. Similarly to 16S sequencing, functional assignment can be biased, due to incomplete reference databases; so far, only up to 50% of all microbial protein sequences may be annotated^16^. Despite remarkable progress in the last decades, developing precise methods for function prediction is still a major challenge in bioinformatics (see CAFA^17^ initiative). The volume of metagenomic data is making the problem even more difficult to deal with. Thus, introducing an in silico method to help contextualize protein functions could prove highly beneficial for realizing the full potential behind metagenomics and multi-omics.

Deep learning is a proven technique for dealing with intricate problems and has been shown to work well for tasks like speech recognition, natural language processing, or image classification^18^. Recently, it has been successfully employed for analysing biological sequences, like genomes, proteomes^19^ or metagenomes^20^. Perhaps the best-known example of the use of deep learning in biology was the protein structure prediction problem. DeepMind's AlphaFold models^21–23^ won the last two Critical Assessment of protein Structure Prediction (CASP) challenges—CASP13^24^ and CASP14^25^, bringing a seismic shift to this decades-old field. The main reason for the notable success of Deep Neural Networks in these areas of biology is their ability to process massive amounts of data, even unlabeled, and extract meaningful patterns from them. Deep learning can leverage the exponential growth of data available in biological databases, which may be limiting for traditional methods. The capability to learn from unlabeled data is particularly valuable due to the constantly increasing gap between the number of unlabeled and labeled protein sequences (https://www.uniprot.org/statistics/TrEMBL).

So far, deep learning methods in protein bioinformatics were employed in two ways: to directly annotate the sequence (supervised learning) or to create a representation of a protein (for example, a sequence embedding using self-supervised learning). Annotation using deep learning is a natural extension of traditional methods, which aim to assign a label to a newly sequenced protein. The label is usually connected to an entry from a database of choice and may belong to curated ontologies (e.g., GO terms^26^) or classification schemes (e.g., EC numbers^27^). Accordingly, studies in the last decade show that deep learning can successfully predict EC numbers^28,29^, GO terms^30–35^, Pfam families^36,37^, or multiple labels at once^38^. However, the labeled proteins are not only in shortage, limiting the potential of deep learning, but also skewed towards model organisms, which may result in biased models.

To overcome these obstacles, more recent approaches use massive unlabeled datasets (UniParc, BFD, Pfam) to train self-supervised models. These models analyse raw amino acid sequences in an alignment-free fashion to learn statistical representations of a protein. The representation can then be effectively used for downstream analyses and predictions of, e.g. secondary or tertiary structure, protein stability, contact map^39,40^, protein function^41,42^, localization^43,44^, variant effect^45^, protein engineering^45,46^, remote homology detection^39^ and more. Moreover, deep-learning-based methods can be used to analyse proteins that do not resemble any catalogued proteins, which is particularly useful in the case of the under-annotated microbiome protein space. Deep-learning-based representations are computationally efficient and accurate, hence they seem appropriate to leverage large amounts of data in high-volume metagenomic studies. However, despite remarkable progress and breakthroughs in several tasks, deep-learning-based approaches are still not mature enough to become prevalent in protein informatics, especially in metagenomics, where further research is needed.

Here, we describe a deep learning approach, based on BiLSTM (Bidirectional Long Short-Term Memory) model^47^, which leverages deep sequence embeddings to understand their potential for solving metagenomic challenges. We trained the model on 20 million microbial proteins from the Unified Human Gastrointestinal Protein (UHGP) catalogue^16^, and then demonstrated the utility of the proposed representations on the Bacterial SwissProt database.

In the first part of this paper, we assessed the type of information encoded in the embedding space and showed that the model built on metagenomics-derived data is more suited for metagenomic applications than Pfam dataset which mostly is a subset of UniProt. In the second part, we visualized and interpreted the space using Uniform Manifold Approximation and Projection (UMAP)^48^, which allowed for a better interpretation of the evaluation results. As an extension, we built an interactive visualization of the space, which is available at https://protein-explorer.ardigen.com. Finally, we present the advantages of the embeddings on an example of short-chain fatty acid kinases.

Overall, deep protein representations show promising potential to become a cornerstone for a new generation of metagenomic tools. A deep model can create a global protein space, strongly related to protein function, by making use of unannotated protein sequences in an unsupervised manner. Representing proteins in this space enables their rapid analysis, using a wide range of traditional methods operating on vector spaces and facilitates tasks, such as classification, clustering, semantic search, or visualization. Accurate projection of protein sequence to a continuous space may even enable research on new methods that were impossible or impractical on the discrete sequence space. The model learns abstract patterns that combine, but also go beyond protein sequence and domain architecture. The use of representation space enables to group even sequentially distant proteins into clusters of proteins sharing similar functions. Model can leverage GPUs to efficiently compute embeddings, and once they are computed, they can be efficiently processed in multiple scenarios (Supplementary Table 6) Still, we are only in the infancy of deep-learning-based metagenomic tools, and further research is needed to fulfill their potential and develop widely-used toolsets.

## Results

### Alignment-free deep protein embeddings represent structure- and function-related ontologies

Metagenomic data may generate an amount of information on the order of tens of millions of reads, which may be assembled into millions of protein sequences. For traditional sequence homology or profile-based approaches, this amount of data is manageable, but requires significant computing power. For deep learning, on the other hand, such a large amount of data provides an opportunity to be exploited for training and assures a robust representation of analysed sequences.

**Figure 1:**
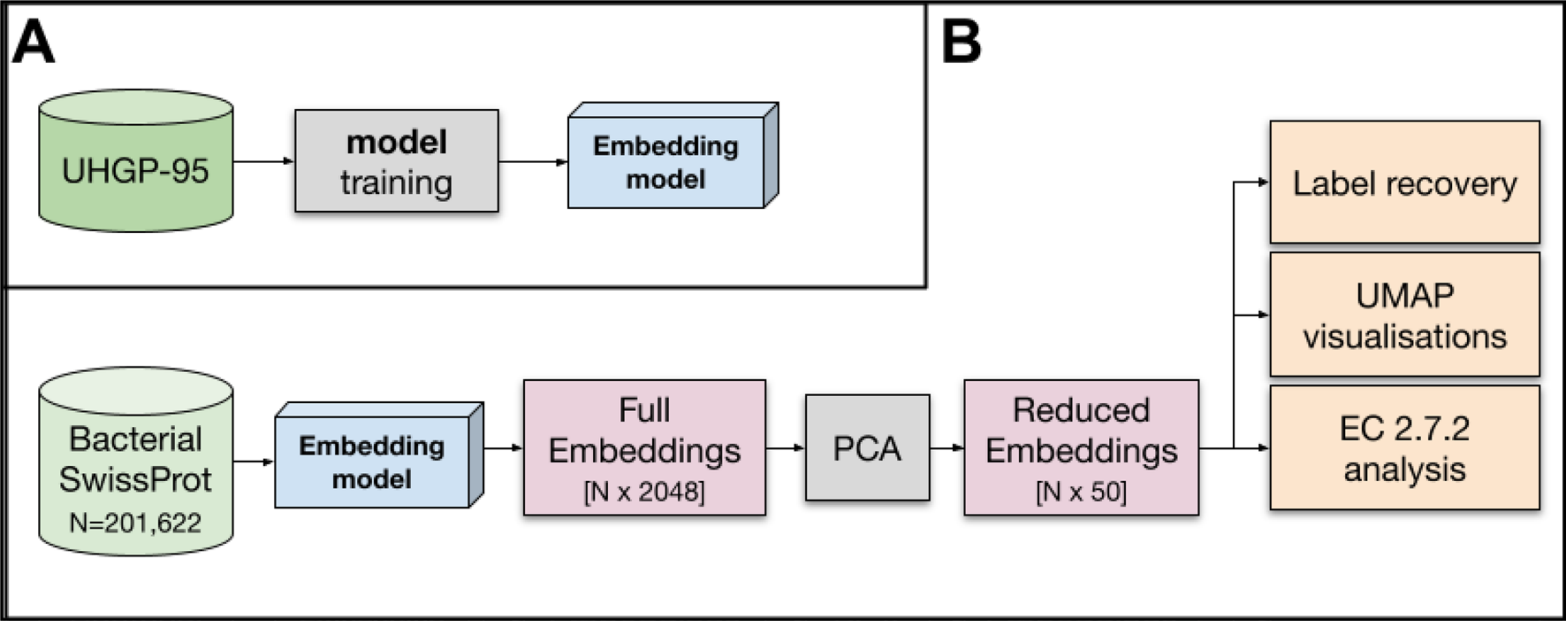
Workflow showing the training of the model and its subsequent use in analyses. (**A**) Training of the embedding model using UHGP dataset. (**B**) Using Bacterial SwissProt dataset and the embedding model to analyse information encoded into the embeddings.

To build the deep representation, we trained the BiLSTM model on the Unified Human Gastrointestinal Protein catalog (UHGP), which contains 625 million microbial protein sequences clustered with MMseqs2 linclust into 20,239,340 representative sequences at 95% amino acid sequence identity^16,49^. From the trained model, we take a hidden-state vector that acts as a protein representation (see “Methods” and Fig. 1A).

Our ultimate goal is to produce reliable embeddings for metagenomic data, hence we first validated the model on proteins derived from ten metagenomic samples that were not included in the UHGP catalog (see “Methods” for details). For that task we chose samples from two metagenomic studies not included in the UHGP dataset (PRJEB37249 & PRJNA762199). From the latter one we selected only healthy volunteers samples to validate results with a healthy human gut microbiome, while the former study (PRJEB37249) focuses on a single enterotype (Bact2) from a Body Mass Index Spectrum cohort The model yielded substantially lower Exponential Cross-Entropy (ECE) loss than the analogous model trained on the Pfam database^50^ on both validating datasets (see Table 1). Although ECE loss does not directly measure the quality of obtained embeddings, it was proven that the lower ECE the better the embeddings are in secondary structure and contact predictions^40^. Pfam is a cross-sectional curated dataset built on top of UniProtKB and is limited to identified protein families (~77% of UniProtKB sequences). The UHGP, on the other hand, is a more comprehensive database for gut metagenomic samples which in many cases (up to ~ 40%) are not represented in protein classification databases (eg. InterPro), and consequently in Pfam^16^. This emphasizes the importance of training an embedding model on a set of proteins consistent with the investigated dataset, i.e. human gut metagenomic proteins. Taken together, this leads to improved model performance.

**Table 1:** Results from metagenomic validation of the trained models. Exponential Cross-Entropy (ECE) measures how good the model is at the training task, which is predicting the next or the previous amino acid in a protein sequence. More detailed results can be found in Supplementary Tables 1 and 2.

Dataset	EBI-ENA Study Accession ID	ECE	
		Model trained on UHGP	Model trained on Pfam
*Bact2* enterotype	PRJEB37249	**10.9** ± 0.4	15.3 ± 0.6
healthy subset	PRJNA762199	**8.5** ± 0.4	13.44 ± 0.2

1,2.

Although the representation is aimed for metagenomic data, we need proteins with a specified function and origin to validate it. Therefore, for our analysis, we used bacterial proteins from the SwissProt database clustered into 201,622 representatives at 97% sequence identity. SwissProt is a reliable source, linking proteins to many ontologies that enable a multilevel description of sequences (e.g. Table 2). For simplicity, we call this collection of proteins Bacterial SwissProt (see “Methods”). We generated embeddings for all Bacterial SwissProt sequences using the embedding model trained on the UHGP dataset. The model trained on Pfam cannot be validated on Bacterial SwissProt as those datasets significantly overlap. Embeddings were then reduced from 2,048 dimensional vectors with Principal Component Analysis (PCA) to 50 dimensions (81.8% of variance explained). Such a representation is used in all our analyses (Reduced Embeddings in Fig. 1B). Rationale for selected parameters can be found in the “Methods” section.

**Table 2:** Description of Bacterial SwissProt ontology databases. For the label recovery task, we used a number of ontologies that can be assigned to a protein. These ontologies are based on 3D protein structure (SUPFAM, Gene 3D), domains (Pfam, InterPro), function (GO, KO, EC numbers) or provide information about organism of origin (taxonomy).

Database	Category	Description	Bacterial SwissProt	
			#Proteins	#Classes
SUPFAM	Structure	SUPFAM associates sequence families from Pfam with SCOP structural families using profile matching to produce sequence superfamilies of known structure	147,137	989
GENE 3D	Structure	GENE 3D contains protein domain assignments for sequences from all of the major sequence databases. Domains are predicted using a library of representative profile HMMs, derived from CATH superfamilies or directly mapped from structures in the CATH database	116,919	1173
InterPro	Sequence and domain	InterPro brings together 11 protein family databases (CATH-Gene3D, HAMAP, PANTHER, Pfam, PRINTS, ProDom, PROSITE Patterns, PROSITE Profiles, SMART, SUPERFAMILY, and TIGRFAMs). Each database provides a specific signature i.e. position-specific score matrices, hidden Markov models and profiles etc. to increase the sensitivity of protein classification	198,677	12,244
KO (KEGG Orthology)	Function	KO is a database of molecular functions. Each molecular function is represented in terms of a manually defined functional ortholog that together create molecular networks (pathways). Each functional ortholog is defined from experimentally characterized genes and proteins in specific organisms, which are then used to assign orthologous genes in other organisms, based on sequence similarity	177,018	6614
GO (Gene Ontology)	Function	GO is a controlled terminology that can be used to consistently and structurally identify genes and gene products. The GO terms are organized within a directed acyclic graph (DAG), and each GO term has a described relationship to one or more other terms in the same domain (i.e. biological process, molecular function, or cellular location)	192,990	5799
eggNOG	Function and taxonomy	eggNOG is a database of orthology relationships, gene evolutionary histories and functional annotations. It is built on the concept of OGs (orthologous groups) that are the result of a non-supervised analysis of thousands of genomes and relationships between all their genes	162,261	15,932
EC number	Function	EC numbers are a manually assigned nomenclature that describes enzymes, based on the chemical reactions they catalyse	193,198	3005
Pfam	Sequence and domain	Pfam is a database of protein families and domains. Each Pfam family has a seed alignment that contains a representative set of sequences for the entry. This alignment is used to build a hidden Markov model profile and the profile is being searched in the sequence database called pfamseq using the HMMER software	120,184	5551
Taxonomy: Order	Taxonomy	Uniprot uses the NCBI taxonomic database to assign taxonomic identifiers to nucleotide sequences	200,536	132
Taxonomy: Family			198,996	274
Taxonomy: Genus			200,615	660

**Figure 2:**
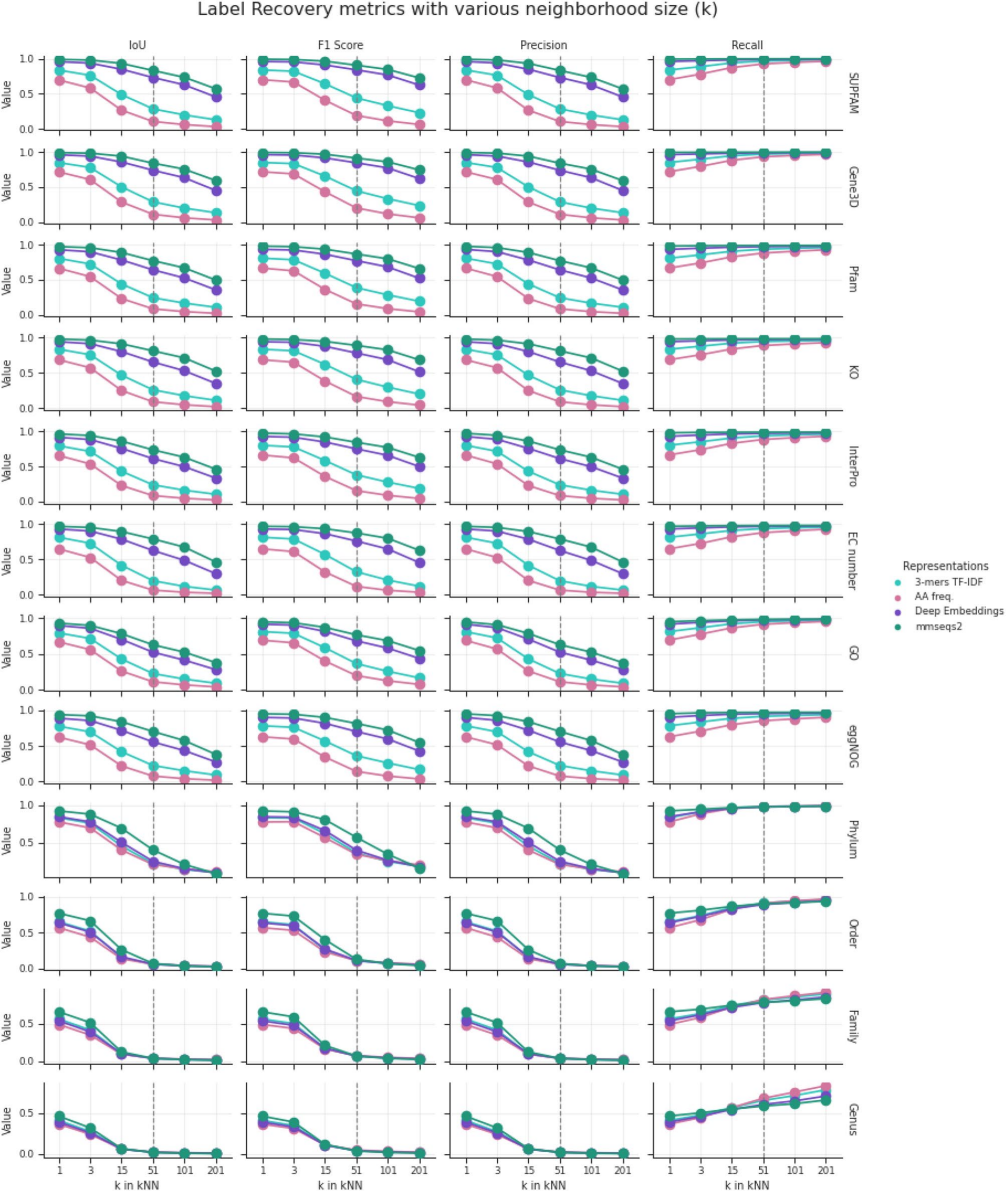
The degree of correctness in the recovery of labels using deep, k-mer-based, and amino acid frequency representations, and MMseqs2—state-of-the-art proteins search tool. The recovery is measured by four metrics: Intersection over Union (IoU), F1 Score, Precision, and Recall. Ontologies are sorted by average results.

To get a deeper understanding of the type of information encoded within deep representations, we created an evaluation task of recovering the label of a given protein from the labels of its nearest neighbors for a cross-section of various ontologies. If the label is correctly recovered, it indicates that the representation is consistent within this ontology (Fig. 2). Using different neighborhood sizes, we can estimate how local the representation is. This study focuses on investigating the representation and its features, not aiming at creating or evaluating a universal label predictor.

To evaluate the consistency of the representation, we selected a number of ontologies from Bacterial SwissProt, related to Function, Structure, or organism of Origin (Table 2). The ontologies significantly vary in the number of classes and Bacterial SwissProt coverage. Hence, the recovery task for each ontology may have a varying degree of difficulty. For this reason, we compared deep embeddings to general scalable sequence-based representations that do not use deep learning. Those baseline embeddings are 3-mers with term frequency-inverse document frequency (TFIDF) transformation^51,52^, and amino-acid frequencies vectors (see “Methods”), similarly to seminal works in this field^37,40,45^. Additionally, we define the upper bound for the task by including MMseqs2 search results, a state-of-the-art tool specifically designed for the protein search task. It should be emphasized that MMSeqs2 does not produce a vector representation and is not versatile as embeddings, which can be used in other types of vector analyses (visualization, clustering, semantic search).

In order to measure label recovery performance of our and baseline representations, we used a cross-validation-based approach. We removed labels of 20% randomly selected proteins in the dataset. Next, we trained a k-Nearest-Neighbor (kNN) classifier. Then, for every protein without a label, we predicted its label based on all k nearest neighbors. We repeated this procedure 5 times for each k.

Many proteins are annotated with more than one label within each ontology (for example, a protein may have multiple Pfam domains). To overcome this challenge, we used the Intersection over Union (IoU) metric and example-based Precision, Recall, and F1 Score metrics^53^.

### Embedding performance on structure-, function- and taxonomy-related ontologies

Despite a varying number of classes in each task, the results from all ontologies unrelated to taxonomy were similar (Fig. 2 and Supplementary Table 3). This suggests a comparable degree of difficulty among them, which we hypothesize that is due to the correlations between labels (e.g. KOs are correlated with Pfam domains). The performance of all methods drops for taxonomic labels, esp. genus, family, and order (Fig. 2). EggNOG ontology, that combines information about function and taxonomy, achieves IoU values that are between those obtained for only function- and only taxonomy-related ontologies. Moreover, baseline representations show that the task's difficulty increases with a larger neighborhood (larger *k*). Despite that, MMseqs2, as a tool designed specifically for the protein search, was able to find similar proteins even from larger neighborhoods. The deep representation results, with a simple kNN classifier on top, were slightly worse in all metrics and ontologies.

The deep representation and MMseqs2 perform best at recovering labels from ontologies based on protein structures (Gene3D, SUPFAM), while function- or domain-related ontologies obtained a slightly lower metric.

MMseqs2 searches for proteins by comparing sequence k-mers in a very clever and efficient way. If two proteins share the same structure or function, even with not so similar sequences in general, they usually share similar sequential patterns that define those functions or structures. MMseqs2 can find those patterns in sequences of both proteins.

However, vector representations presented here work differently. They produce a vector summarizing the whole protein sequence. Baseline representations (3-mers with TFIDF and amino-acid frequencies vectors) treat different parts of a sequence with equal importance, so the essential sequential patterns are lost in the burden of many neutral mutations. On the contrary, the deep model during the training can learn that some sequential patterns often occur in a training dataset with only minor changes (conserved regions) and have the most significant impact on the rest of the sequence^54^. During the process of embedding a sequence, the model can put significantly more attention on those sequence fragments. This way deep embeddings can contain essential information to obtain results comparable to MMseqs2 on function- and structure-related ontologies without directly comparing the sequences.

The taxonomy case is different (Fig. 2 bottom 4 panels). Proteins with the same organism of origin still can share sequential patterns that can be found using MMseq2 search. However, the deep model will not focus on those motifs, as they neither occur often in the training dataset nor have substantial impact on the rest of the sequence. They may have a marginal impact on the deep embedding, and so it will not have any advantages over baseline representations.

These results indicate that the deep representation space encodes features related to protein structure and function^55^, and does not represent features related to the taxonomy.

Despite not achieving the highest metrics, we recognize deep embeddings as a very promising method combining advantages of a top end sequence-based tool (effectiveness in finding functionally similar proteins) and vector representations (versatility and efficiency once they are computed; Supplementary Table 6). Future developments of the approach may boost both effectiveness and efficiency.

### Low dimensional representation of protein sequence space goes beyond sequence similarity

Representations learned by deep models are information-rich, but more difficult to understand due to the high dimensionality of the embedding. Further reduction in dimensionality with UMAP allows us to plot and visually interpret the embedding space built by the model.

### Deep embedding model creates a functionally structured representation space

**Figure 3:**
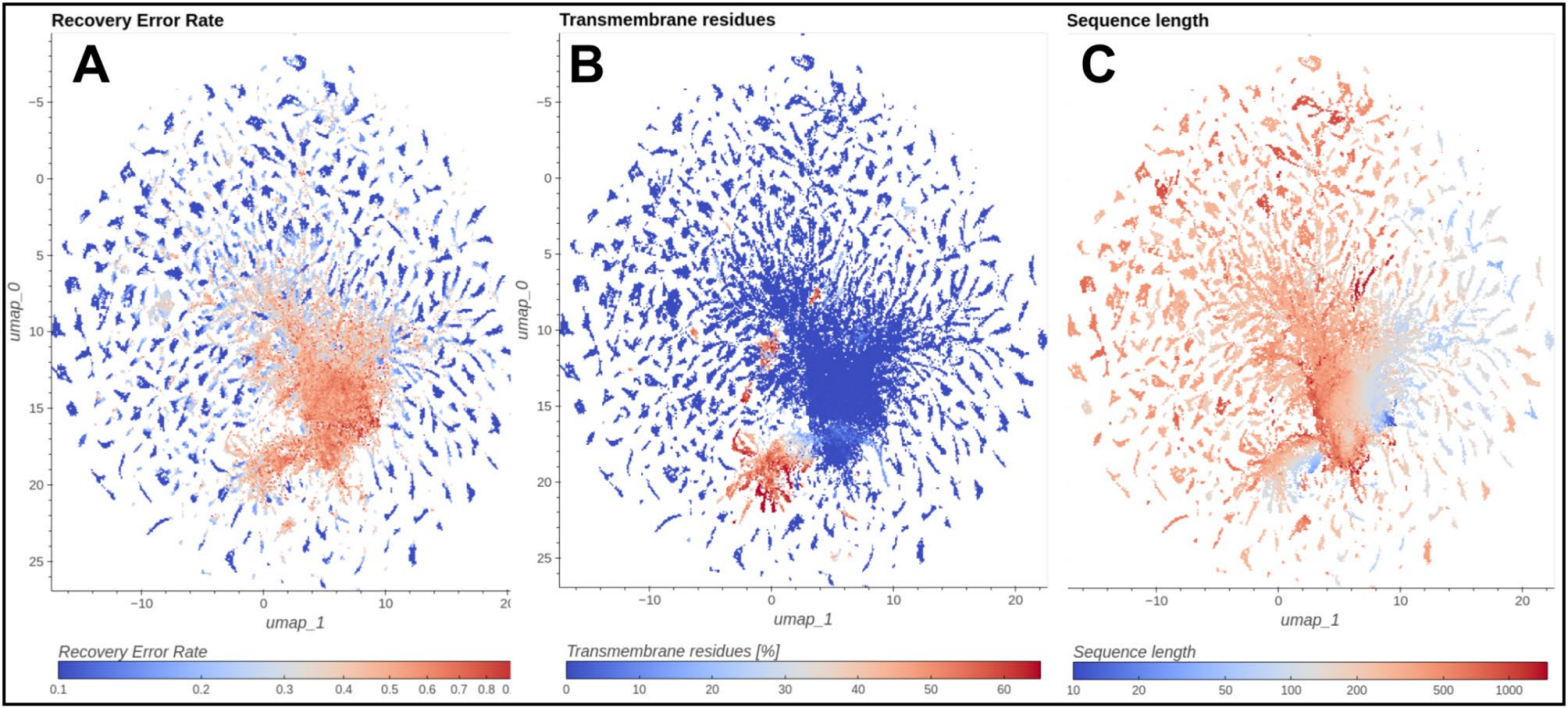
Visualization of the first two UMAP components of Bacterial SwissProt embeddings. (**A**) Proteins colored by Recovery Error Rate, the metric that quantifies how hard it was to recover protein’s labels based on its neighbors, the metric that quantifies how hard it was to recover protein’s labels based on its neighbors. (**B**) Proteins colored by percentage of transmembrane residues in a protein chain; adopted from Perdigão et al.^56^. (**C**) Proteins colored by sequence length.

To better understand which proteins were the easiest to recover based on the embedding, we defined Recovery Error Rate as *1—average IoU* metric obtained on each protein across all ontologies and all k values. The use of this metric enabled us to localize regions with low & high Recovery Error Rates (Fig. 3). In Fig. 3A, we show that proteins with low Recovery Error Rates are located in smaller clusters, while proteins with high error rates are concentrated in the center of the UMAP visualization.

**Figure 4:**
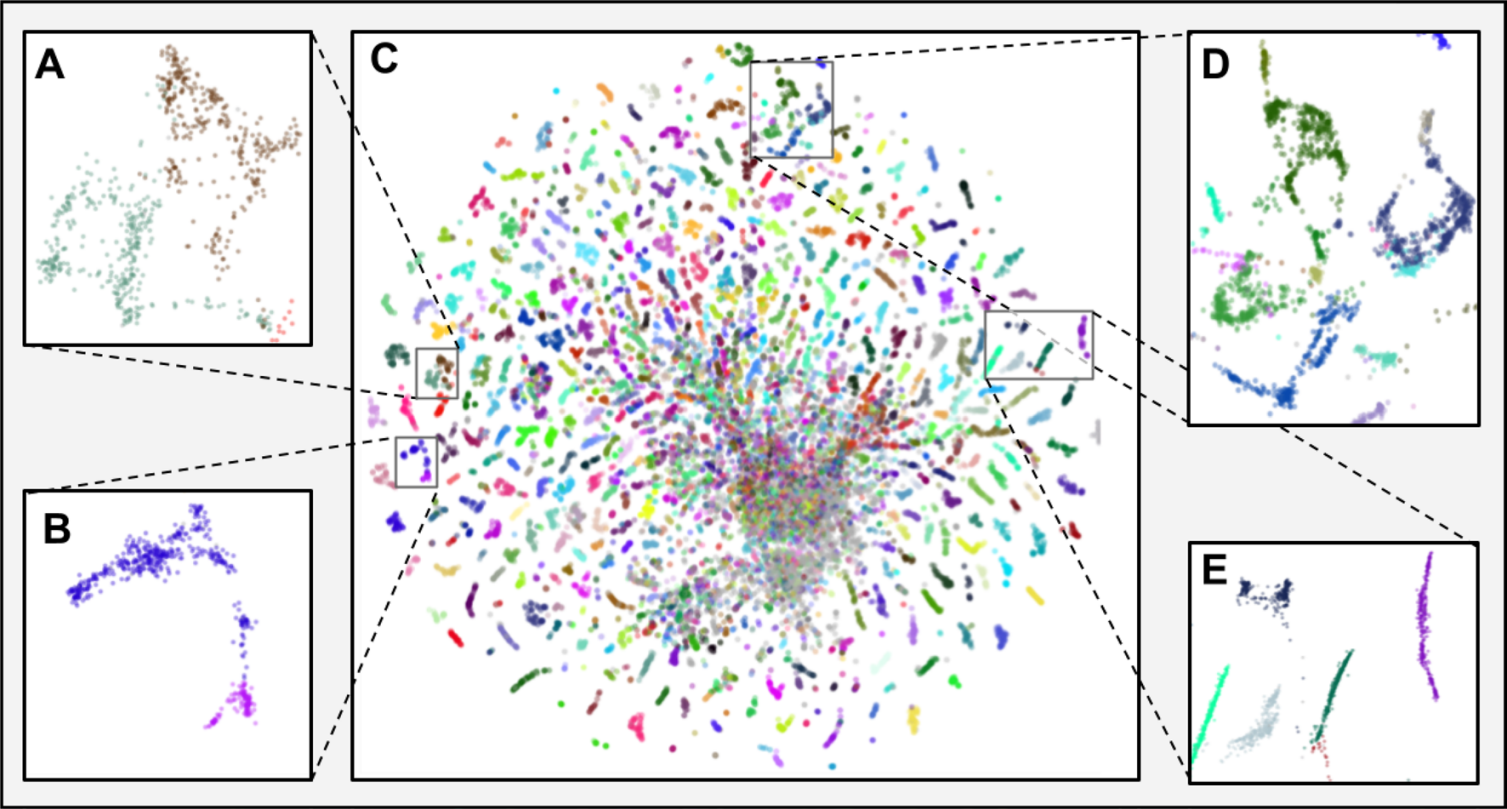
Deep embeddings UMAP projection of Bacterial SwissProt colored by KO. **(A)** transferase proteins that share the same Pfam domain and belong to the EC 2.5.1 class—UDP-N-acetylglucosamine 1-carboxyvinyltransferase (K00790) in dark green, 3-phosphoshikimate 1-carboxyvinyltransferase (K00800) in brown. **(B)** GTP binding proteins sharing Pfam domains—Elongation Factor G (K02355) in purple, Peptide chain release factor (K02837) in pink. **(C)** All Bacterial SwissProt proteins. **(D)** proteins that belong to the tRNA ligases class (EC 6.1.1)—Cysteine (K01883) in dark green, Arginine (K01887) in blue, Glutamate (K01885) in navy blue, Glutamine (K01886) in cyan,Valine (K01873) in pink, and Isoleucine (K01870) in light green. **(E)** ribosomal proteins—30S ribosomal protein S1 (K02961) in light green, 50S ribosomal protein L14 (K02874) in light blue, 50S ribosomal protein L36 (K02919) in black, 50S ribosomal protein L35 (K02916) in dark green, and 50S ribosomal protein L15 (K02876) in purple.

To investigate the functional structure of the representation space, we overlay it with labels defined by Kegg Orthology ID (KO) (Fig. 4). The proteins that do not have a KO assigned are colored in grey—we see that they are placed in the central part of the plot. Most of the proteins are clearly clustered by their functional annotation. Furthermore, by focusing on specific space locations, we can see that close KO clusters share other functional features: domains (Fig. 4A,B), EC number class (Fig. 4A,D), or structural and molecular features (Fig. 4E). It suggests that the deep representation does not focus only on one functional ontology, but rather on an abstract protein function defined on many levels. The visualisation explains the high label recovery results and expands analogous analysis conducted on a smaller scale with only 25 COGs^40^. Compared to the k-mer based representation, the deep representation is significantly more structured (Supplementary Fig. 1).

We hypothesize that the regions of high Recovery Error Rate (RER) are occupied by rare proteins. Rare proteins form small functional classes in Bacterial SwissProt, and the smaller the functional class is, the more difficult it is to predict the label based on its neighbors. Additionally, their potential insufficient representation in the training set makes it difficult to model their sequences, as the embedding model can learn certain patterns only if they are shared by a sufficient number of proteins in the training dataset. Indeed, we observed that the Recovery Error Rate is negatively correlated (r = − 0.776, N = 200,115) with the log-average size of the functional class the protein belongs to (See Supplementary Fig. 2). Moreover, we noticed an increased frequency of the occurrence of words: *‘Uncharacterized’*, ‘*Putative*’ and ‘*Probable*’ in SwissProt descriptions of high RER proteins (43.2% for Recovery Error Rate = 1 vs. 1.2% for Recovery Error Rate = 0, See Supplementary Fig. 3), indicating less characterized proteins. Indeed, an analysis of KEGG categories as a function of RER seems to corroborate this (Supplementary Fig. 4). We see that high RER proteins generally belong to smaller classes and are responsible for more varied functions. For example, *transcription* and *translation* KEGG categories exhibit low RER, while *Protein families: metabolism* or *Biosynthesis of other secondary metabolites* show high RER. The latter, is a large category consisting of many pathways involved in biosynthesis of phytochemical, antibacterial, fungal, and other compounds. Overall, analyses show that groups with high mean RER are more diverse or are rich in proteins that are rare or less described in databases than groups with low mean RER (Supplementary Figs. 2, 3 and 4).

### Short and transmembrane proteins

The embedding model is sensitive to the length of the protein (Fig. 3C) and a significant number of short proteins is present in the central, lesser understood part of UMAP visualization. Short proteins (≤ 50 residues), underestimated for a long time, gained interest in recent years when it was discovered that they are involved in important biological processes such as cell signaling, metabolism, and growth^57^. The presence of a high Recovery Error Rate region might be a result of insufficient information on small proteins, which are still underrepresented in databases. Following Sberro et al., based on the NCBI GenPept database, over 90% of small protein families have no known domain and almost half are not present in reference genomes^58^.

For a detailed description of the protein set see Supplementary Table 4. The whole space can be interactively explored in our application (https://protein-explorer.ardigen.com).

Transmembrane proteins constitute approx. 30% of all known proteins. Unlike globular proteins, they are on average larger and must exhibit a pattern of hydrophobic residues to fit into the cell membrane^59^. In order to define transmembrane proteins we used a transmembrane score (a percentage of transmembrane residues) adopted from Perdigão et al.^56^. In Fig. 3B, we can see that the model separates transmembrane proteins well, which is in line with previous research on deep protein representations^44,60^. However, part of transmembrane proteins lie within the high-recovery error region of the UMAP plot. Despite substantial pharmacological and biological relevance, they are less understood and underrepresented in databases, as structural experiments on them are difficult to conduct.

Lower ECE loss obtained on metagenomic proteins (compare *Alignment-free deep protein embeddings represent structure- and function-related ontologies* section) suggest that the deep embedding model trained on a more general catalog of metagenomic proteins (UHGP) is less biased towards well-known model organisms, hence, better suited for rare, short or transmembrane proteins.

### A sample use case—phosphotransferases (EC 2.7.2)

To demonstrate the use of embedding representation in a real-life scenario, we used a group of phosphotransferases. We have chosen them due to their importance in maintaining the human gut microbiome homeostasis. Acetate, butyrate, and propionate kinases are especially crucial in the process of forming short-chain fatty acids (SCFAs). SCFAs are produced in the colon by bacteria during the fermentation of resistant starch and non-digestible fibers. Their lowered level is often observed in patients suffering from inflammatory bowel diseases (IBD) such as Crohn’s disease and ulcerative colitis^61^. SCFAs serve as an important fuel for intestinal epithelial cells and participate in preserving gut barrier integrity. Recent findings indicate their role in energy metabolism (lipid metabolism), immunomodulation, regulation of intestinal epithelial cells, proliferation and cancer protection. Although promising, the research has been conducted mainly on murine or in vitro models, thus the results have to be interpreted with caution^61–63^.

Proteins classified as phosphotransferases were chosen based on their EC number. We decided to use this annotation as EC numbers are a manually assigned nomenclature that describes enzymes based on the chemical reactions they catalyze. Their hierarchical structure allows for a fine-grained analysis. Proteins described by EC 2.7.2 class represent phosphotransferases with a carboxyl group as an acceptor. We used eight EC 2.7.2 subclasses available at Bacterial SwissProt: EC 2.7.2.1 (acetate kinase), EC 2.7.2.2 (carbamate kinase), EC 2.7.2.3 (phosphoglycerate kinase), EC 2.7.2.4 (aspartate kinase), EC 2.7.2.7 (butyrate kinase), EC 2.7.2.8 (acetylglutamate kinase), EC 2.7.2.11 (glutamate 5-kinase) and EC 2.7.2.15 (propionate kinase).

We examined the domain architecture of EC 2.7.2 proteins using the Pfam database. The domain architecture is the main structure that defines a protein's function. We found that four domain architectures were dominant among analysed proteins. 31% of analysed proteins contained one amino acid kinase domain (PF00696), 29% of proteins had one phosphoglycerate kinase domain (PF00162), 20% contained one acetate kinase domain (PF00871), and 18% of proteins had two coincident domains PF00696 & PF01472, i.e., amino acid kinase domain and PUA domain.

In total, we studied 1,302 proteins exhibiting eight unique specific functions (ECs) and four distinct domain architectures (See Supplementary Table 5). Different domain architectures suggest that these proteins have different amino acid sequences and would be difficult to identify as similar with baseline bioinformatic methods based on sequence similarity alone.

**Figure 5:**
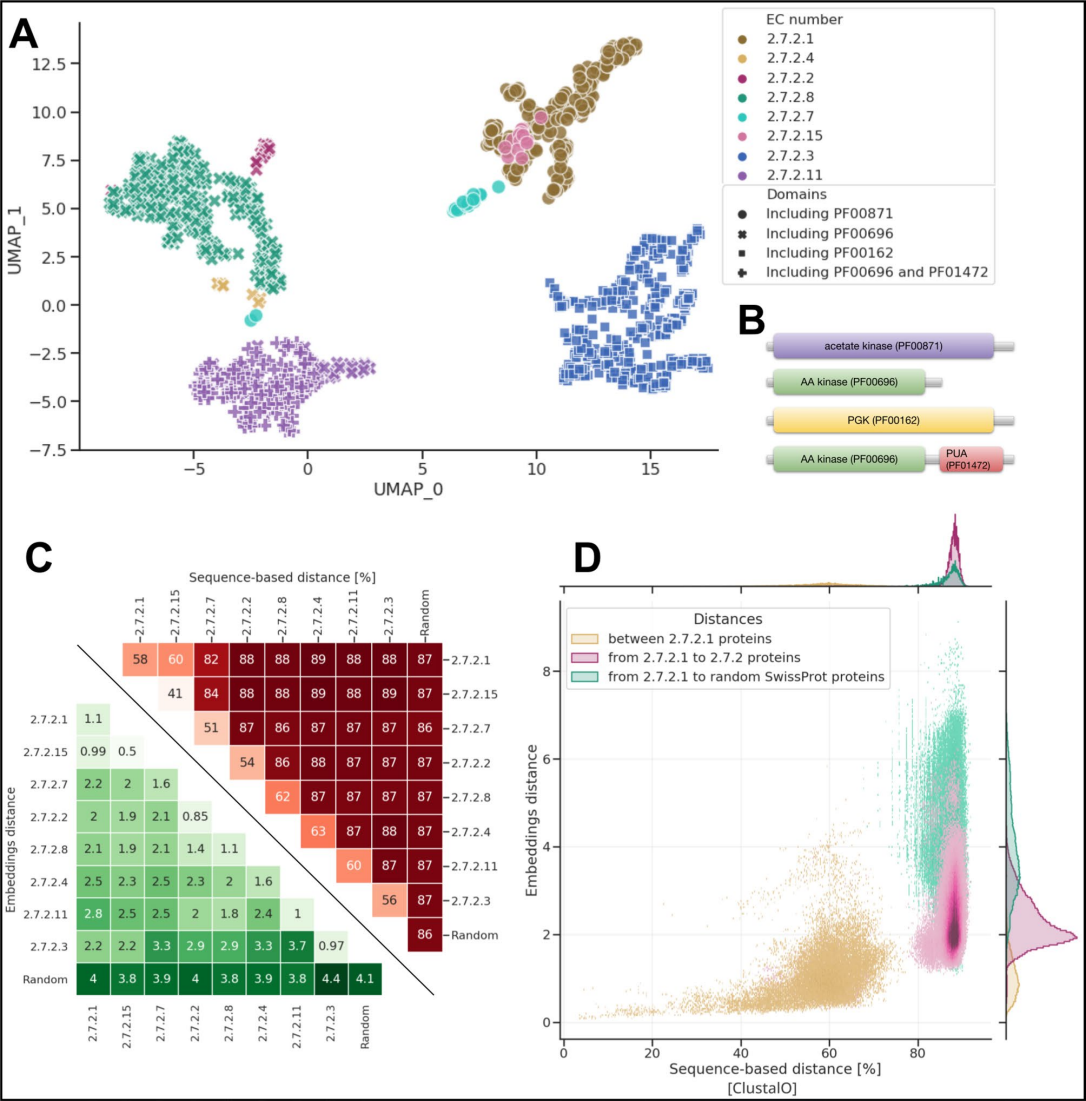
Visualization of EC 2.7.2 proteins in the deep embedding space. (**A**) Deep embeddings of EC 2.7.2 proteins visualized with UMAP. Colors correspond to EC numbers and shapes to PFAM domains. Axes represent UMAP's first two components. (**B**) Domain architecture of EC 2.7.2. (**C**) The mean distance between EC 2.7.2 proteins and 500 random proteins from the SwissProt space with distinction between embedding-based distance (green) and ClustalO distances (red). Values for both methods were calculated as averages of pairwise distances between all proteins within given clusters. (**D**) Comparison of embedding-based and sequence-based distance (ClustalO) to EC proteins 2.7.2.1. The distances were divided into those within the protein group EC 2.7.2.1, from EC 2.7.2.1 to other EC 2.7.2 proteins, and from EC 2.7.2.1 to randomly selected proteins. The embedding-based, as opposed to the sequence-based distance, differentiates the distances from EC 2.7.2.1 to other members of EC 2.7.2 and from EC 2.7.2.1 to random proteins. Marginal histograms represent data distribution of the two analysed distances in three different categories described above.

To investigate how accurately the embedding representation reflects the functional relationships between the proteins, we visualized them using UMAP (Fig. 5A). We understand that both domain architecture and enzymatic activity impact protein embeddings and their ordination in UMAP space. Almost all proteins were grouped according to their domain architecture, and proteins with similar domain architectures, such as proteins having only PF00696 domain and proteins having two domains PF00696 & PF01472, were also placed closer to each other. Despite clear domain-based grouping, proteins that share the same domain architecture, but catalyze different chemical reactions, are separated. We hypothesize that protein domain architecture has a stronger influence on the embeddings than EC number (Fig. 5A,B) as proteins with different domain architecture form clear clusters in UMAP visualization and proteins with only PF00696 domain are clustered closer to proteins that contain both PF00696 and PF01472 compared to proteins with different protein domains. The only exceptions are EC 2.7.2.1 and 2.7.2.15. One possible explanation for this exception is that these two enzymes can share substrates for their activity. Acetate kinases (EC 2.7.2.1) can accept propionate as an alternative substrate, and propionate kinases (EC 2.7.2.15) can accept acetate. Moreover, both EC 2.7.2.15 and EC 2.7.2.1 play essential roles in the production of propionate in bacteria^64^. The only inconsistency we can note are two butyrate kinase proteins (Fig. 5A cyan circles; PF00871) that were placed far from their counterparts.

To further analyse two outlying butyrate kinases, we inspected sequences of outlier’s domains sequence and compared it to sequences of PF00696 and PF00871 domains. We performed multiple sequence alignment (MSA) of outlying protein’s domains, the PF00696 domains and the PF00871 domains sequences (see “Methods”). MSA results showed that outlying protein’s domain sequences are distant from PF00871 domain sequences from further proteins, including other butyrate kinases. However, outliers were more closely aligned with sequences that belonged to PF00696 domains (Supplementary Data 1–3). We hypothesize that the domain sequence of the two outlying proteins is more similar to PF00696 domain than PF00871, which made UMAP place them closer to the former (Fig. 5A,B).

We hypothesize that the deep representation reflects the functional similarities between proteins that are based on domain architecture (Pfam domains) or enzymatic activity (EC number). This emphasizes the significant advantage of deep embeddings, as they do not only focus on single, human-created ontology, such as e.g., EC numbers, but rather fuse all information to characterize proteins on multiple levels. It combines the strengths of approaches that focus on motifs, domains (Pfam), and 3D structure (GENE 3D) to understand protein function space comprehensively. To better understand the differences between sequence-based distance and deep embeddings, we compared the Euclidean distance between EC 2.7.2 proteins and randomly chosen 500 proteins from the Bacterial SwissProt dataset. As a baseline, we selected sequence-based distance calculated with Clustal Omega^65^. The distance measure used by Clustal Omega for pairwise distances of unaligned sequences is the percent identity between two analysed sequences (see “Methods”).

The embedding-based distances within and between the EC 2.7.2 subclasses are smaller than to randomly selected proteins, which do not hold for the sequence-based distance (Fig. 5C,D). The mean embedding-based distance between EC 2.7.2 proteins is significantly smaller compared to the distance between EC 2.7.2 proteins and 500 random proteins. Not only proteins from the same cluster group are closer to each other but also proteins from different EC 2.7.2 clusters are located significantly closer to proteins from other EC 2.7.2 clusters than to random proteins. Mean percent identity between proteins does not reflect the clear separation between EC 2.7.2 and random proteins. Mean sequenced-based distance between proteins from the same cluster is smaller than between EC 2.7.2 and random proteins, however it does not bring proteins closer from different EC 2.7.2 clusters (Fig. 5C). This proves that the embedding model can go beyond sequence similarity and find relations between proteins with significantly different sequences and domain architectures.

We believe that deep protein embeddings may enable searching for proteins that are functionally similar at different levels of specificity. Taking EC number classification scheme as example, while localizing the searched protein sequence in deep embeddings space we can find a cluster of proteins that belong to a general assemblage of transferases (EC 2.7) and splits into more specific subclusters such as EC 2.7.2 (see Fig. 5 and our application). We expect that this will help functionally define new, undiscovered bacterial proteins that implement similar functions (e.g. novel 2.7.2 subclass) with a significantly different sequence.

## Discussion

The human microbiome plays a crucial role in human health, and changes in its composition can be related to various diseases, such as diabetes, cancer, or psychiatric disorders. To fully understand the complex relation between the microbiome and human health, it is necessary to look not just at the taxonomic level but also at a functional level. Despite various approaches to retrieve protein functions^66,67^, a large portion of microbial proteins remain functionally uncharacterized. This paper presents a novel context for using the Bidirectional LSTM model to visualize and contextualize the microbial protein space. We show that our model accurately represents protein features related to structure and function, overcoming some limitations of standard bioinformatics methods such as HMMER or BLAST. However, more research and development is needed to establish deep-learning-based tools that will take them over.

The deep learning model creates an abstract, numerical representation of proteins in an embedding space. This embedding encodes information from various protein ontologies and combines knowledge on protein structure and function, overcoming the limitations of methods based on sequence similarity. For certain tasks processing embeddings is more efficient then sequences, although generating embeddings is still computationally expensive. The embedding is also more suitable for a large range of further downstream algorithms, such as classification, clustering and visualization. Combining embeddings with a dimensionality reduction method, such as UMAP, may enable creating a reference protein map and facilitate protein research.

One of the significant challenges that any data-driven solution must face is data bias. Our results indicate that using a catalog of metagenomic proteins (UHGP) for training made the model less biased towards well-known model organisms. Despite this, model validation required the use of experimentally verified data, which limited the scope of our validation to well-known proteins and prevented genuine validation on small or transmembrane proteins. We assume that with the growing interest in these proteins, their presence in the databases and number of their annotations will increase, which will allow for a more thorough validation.

We are witnessing rapid progress in both the deep learning field and in metagenomics, which generate massive amounts of data. We believe embedding models are an attractive alternative to database-bound, computationally intensive methods unsuitable for such influx of data. We also assume that recent advances in deep learning, like the latest intensive research on Transformer-based architectures, will only improve results presented in our work. Other appealing approach would be to join the strengths of relatively computationally-cheap embedding models with other computational technologies that can accurately predict the features of individual genes (for example: protein 3D structure using AlphaFold^21–23^) and finally perform experimental validation on most promising targets. Such approach enables such efficient contextualization of metagenomic data and may be used to better understand the microbiome for health. Finally, we hope that the research presented here and in other related works will lead to concrete tools that will enable adoption of the approach in microbiome and other metagenomics studies.

## Methods

### Embedding model training

In the training, we took advantage of the Unified Human Gastrointestinal Protein catalog clustered at 95% sequence identity (UHGP-95) to limit the impact of the most common sequences. Further clustering may improve the model^40^. UHGP-95 contains exactly 19,228,304 protein sequences, from which we randomly selected 5% to track training progress (validation set) and set aside another 5% for the final model evaluation (test set). The rest of the data (18,266,888 sequences) was used to train the model. Due to GPU memory limitations we clipped all proteins to 1,500 amino acids. This impacted only 0.9% of proteins from the training set as the others were shorter.

We used a 3-layered Bidirectional LSTMs (BiLSTM) model with 1024 hidden units in each layer. The LSTM-based architectures are relatively well established in the protein informatics, being applied to predict, i.a. subcellular localization^47^, secondary structure^68^ or protein crystallization^69^. Moreover, we have chosen the LSTM architecture as it gave the best results in Remote Homology detection in the TAPE benchmark^39^ and achieved superior performance over Transformer-based architecture in the ProtTrans benchmark^44^. On the other hand, the most recent findings show the superiority of Transformer-based architectures^23,40,70^ in protein informatics. We assume that those and even further advances in deep learning, especially applied to protein sequences, will only improve results presented in our work.

The model was trained by the AdamW optimizer for 225,331 weights updates with a mini-batch of size 1024, which corresponds to 12 epochs and approximately 48 h on 4 Tesla V100 GPUs. The learning rate was set to 1e-3, except the first 8,000 steps that were used as a warmup. The process was implemented in the PyTorch library^71^, based on the TAPE benchmark^39^ repository (https://github.com/songlab-cal/tape).

### Computing embeddings

To obtain a vector representation of a protein (embedding) from the BiLSTM model, we extracted vectors of hidden states for each amino acid and averaged them. This is in contrast to natural language processing practice, which uses the hidden state vector corresponding to the last word (here it would be the last amino acid) rather than the average representation of all words. However, there is evidence suggesting the superiority of averaged presentation in the field of protein processing^45^. This may be due to the fact that proteins are usually much longer than sentences, and LSTM-based models cannot fit the whole amino acid sequence in just one state.

### Validation on metagenomic proteins

The UHGP catalog contains data publicly available as of March 2019, thus for the validation we have selected ten samples each from 2 studies published after that date (See Supplementary Tables 1 and 2; PRJEB37249 and PRJNA762199)^72^. From the second dataset, we chose only samples from healthy volunteers, as indicated by the authors of the study (not yet published, available at PRJNA762199). We assembled samples using MEGAHIT v1.2.9^73^ (megahit -1 {sample}_1.fastq -2 {sample}_2.fastq -o megahit/{sample} -t 1ʘ -m 2ʘ48ʘ) and retrieved protein sequences using prodigal v2.6.3^74^ (prodigal -i {sample}_final.contigs.fa -a prodigal/{sample}.faa -p meta) on obtained contigs. We measured ECE loss on each sample separately and averaged values to obtain final results. For comparison we trained a model on Pfam database v32^75^—the same as used in TAPE benchmark^39^. The model architecture and the training process were the same as in the UHGP model training described above. However, changing the training dataset resulted in 32,207,059 training sequences, 401,543 weights updates, and 59 h of training.

### Bacterial SwissProt

For evaluating the properties of the embedding space, we used the UniProtKB/Swiss-Prot 2019_02 database with 562,438 protein entries. For every entry, we parsed taxonomy lineage and functional labels (Table 2). Only proteins from the Bacteria domain were selected, leaving 331,523 proteins.

To remove near-identical protein sequences, we deduplicated the remaining set using *mmseq2 easyclust*^76^ with an identity threshold set to 97% and coverage set to 0.8 (mmseqs easy-cluster uniprot_sprot.fasta swiss97_clust tmp -e inf -c ʘ.5 --min-seq-id ʘ.1 --cov-mode 1 --cluster-mode 1 --threads 20). Removing duplicates ensured no cliques in the kNN graph, which we used in the kNN label recovery and UMAP visualizations. Cliques would lead to trivial solutions during kNN classification and “lonely islands” in UMAP visualizations.

After the deduplication step, we obtained 201,622 proteins, and this set we named Bacterial SwissProt.

### Baseline representations

For a general sequence-based baseline representation, we used the bag of k-mers method^77^, which produces embedding for a protein by the following procedure: (a) generate all possible k-mers (subsequences of length k) from protein sequence, (b) count occurrences of each possible k-mers in the sequence, (c) sort counts alphabetically by k-mers sequence. Sorted counts form a vector representing the sequence.

Higher k leads to more specific representation but exponentially increases dimensionality, which is equal to the number of all possible k-mers (N = 20^k^). In our work, we chose k = 3, which resulted in 8,000-dimensional vectors. Choosing k = 4 would lead to 160,000 dimensions, which would be hard to manage computationally. On the other hand, k = 2 would be convenient with 400 dimensions but less specific than k = 3.

Finally, we have applied term frequency-inverse document frequency (TFIDF) transformation on the 3-mer representation, which accentuates rare k-mers. We have used sklearn’s TfidfTransformer^78^ to implement the transformation.

To complement the k-mer-based baseline we added a representation of the amino acid frequencies vector.

### MMseqs2 search baseline

For a task-specific, state-of-the-art baseline we have used MMseqs2 search^76^. It is a fast and sensitive sequence search tool that is broadly applied in metagenomics. We needed to increase MMseqs2 search sensitivity (from default 5.7 to 9.0) and suppress e-value thresholding to obtain up to 201 nearest proteins from the search. These are not the best parameters if one is looking only for the several nearest proteins, but it was necessary to compare larger neighbourhoods. Full commands are listed below.
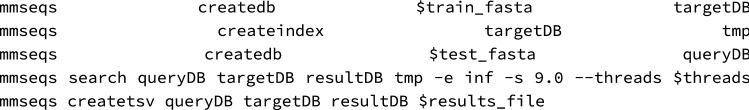


### Label recovery

For the analysis, we used the Bacterial SwissProt described above. We generated deep and k-mer-based representations for each protein. Next, we reduced the dimensionality of all representations to 50 using the Principal Component Analysis (PCA) algorithm (Fig. 1B). Vectors of 50 dimensions are computationally efficient for downstream analyses and at the same time explain 81.8% of variance of the full embeddings.

We narrowed down the set of analysed proteins to only those with assigned labels in given ontology for each ontology analysed. We divided these sets of proteins into five equal parts to estimate recovery efficiency through fivefold cross-validation. For every fold, we constructed a kNN graph (https://github.com/lmcinnes/pynndescent) of the data from the four remaining folds. The graph was then used to predict classes for each protein in the fold, by querying the nearest proteins (N = 51) and propagating their labels as a prediction. As the protein can be assigned to many classes (multi-label classification), we used the Intersection over Union (IoU) as the main metric. IoU is the ratio between the correctly predicted labels and the union of all predictions with all ground-truth labels for a given protein (1). IoU ranges between 0 and 1, where 1 means perfect label recovery. For single-label tasks, IoU reduces to accuracy.

1 $$IoU = \frac{| prediction \cap ground-truth |}{| prediction \cup ground-truth |}$$

We also included example-based Precision (2), Recall (3), and F1 Score (4) metrics^53^.

2 $$Precision = \frac{| prediction \cap ground-truth |}{| prediction |}$$

3 $$Recall = \frac{| prediction \cap ground-truth |}{| ground-truth |}$$

4 $$F1 Score =2 \frac{Precision * Recall}{Precision + Recall}$$

### UMAP visualisations

To visualise protein embedding space, we further reduced dimensionality of the PCA Embeddings with UMAP^48^ (Uniform Manifold Approximation and Projection; https://github.com/lmcinnes/umap), a nonlinear dimensionality reduction method. UMAP was chosen over another common nonlinear dimensionality reduction method, t-SNE (t-distributed Stochastic Neighbor Embedding), as it preserves more of the global structure with superior runtime performance^79^.

We set the number of neighbors (*n_neighbors)* to 50 to balance representing the local and global structure of the data. Also, we set the minimal distance (*min_dist)* to 0.3 to ensure the visibility of all proteins on the scatterplots. The rest of the parameters were left at default values.

#### Cluster analysis

##### Selecting proteins

Proteins assigned to EC 2.7.2 subclass were chosen for the analysis. In the analysis, we used 8 available EC 2.7.2 sub-subclasses out of 14, as our bacterial dataset lacked proteins described by 6 other sub-subclasses. Sub-subclasses used in this analysis are EC 2.7.2.1 (acetate kinase), EC 2.7.2.2 (carbamate kinase), EC 2.7.2.3 (phosphoglycerate kinase), EC 2.7.2.4 (aspartate kinase), EC 2.7.2.7 (butyrate kinase), EC 2.7.2.8 (acetylglutamate kinase), EC 2.7.2.11 (glutamate 5-kinase) and EC 2.7.2.15 (propionate kinase). We assigned a Pfam ID to each protein using mapping available in SwissProt. 4 domain architectures were found dominant among 1,302 analysed proteins. 31% of analysed proteins contained one amino acid kinase domain (PF00696), 29% had one phosphoglycerate kinase domain (PF00162), 20% one acetate kinase domain (PF00871) and 18% had two coincident domains (PF00696 and PF01472), i.e. amino acid kinase domain and PUA domain.

We visualized EC 2.7.2 proteins in the same manner as described above in *UMAP visualizations*.

##### Comparison to sequence (Clustal Omega for distance matrix)

To infer about the ability of the embedding model to group more closely proteins sharing a function, we compared the distance between EC 2.7.2 proteins and 500 randomly chosen proteins from the Bacterial SwissProt database (excluding EC 2.7.2 proteins). We wanted to analyse if embeddings distance between proteins is compatible with corresponding amino acid sequence distance. Embedding distance was calculated as an Euclidean distance between 50 PCA components. Those 50 PCA components are the result of dimensionality reduction of 2048 protein embeddings, generated by the model. Sequence distance was calculated using Clustal Omega^80^ (clustalo --infile $sequence_file --seqtype = Protein --distmat-out $distance_matrix -clustering-out = $clustering --outfile = $alignment --threads = 16 --percent-id --full), a bioinformatic tool for multiple sequence alignment. This tool takes a fasta file with unaligned protein amino acid sequences as input and calculates percent of sequence identity between those sequences giving a pairwise distance matrix. The distance measure used by Clustal Omega for pairwise distances of unaligned sequences is percent identity between two analysed sequences.

We have chosen to draw 500 proteins to have a big enough sample and at the same time limit required computations (the number of distances to compute grows quadratically with the number of proteins). Results were almost identical when we drew 100, 1000, or different 500 proteins. We believe that in this case, 500 proteins are enough to model the distribution of “other proteins”. The selected 500 proteins are listed in the notebook on our Github repository (https://github.com/ardigen/microbiome-protein-embeddings/blob/master/03-ec-2.7.2/ec-2.7.2-analysis.ipynb).

##### Outliers analysis

To infer sequence similarity we performed multiple sequence alignment (MSE) between outlying protein’s, PF00696 and PF00871 domain sequences. HMMER software^67^ was used to find domain positions in each protein. First we created a hmmer profile database using target domains (hmmbuild $hmm_database $alignment_file, hmmpress $hmm_database) and searched domains in outlying proteins (hmmscan $hmm_database --tblout -E 1e-5 $searched_proteins_seq_file > out $output). Biopython 1.79^81^ was used to extract domain from protein sequence. MSE was performed using Clustal Omega^65^. We performed three MSE: (a) outlying butyrate kinases vs other butyrate kinase proteins, (b) outlying butyrate kinases vs PF00696 domain sequences from proteins containing that domain, (c) outlying butyrate kinases vs PF00871 domain sequences from proteins containing that domain. We visualized the alignment using Jalview^82^ (using default color scheme used for alignments in ClustalX).

## Supplementary Information

Supplementary Information 1.

Supplementary Information 2.

Supplementary Information 3.

Supplementary Information 4.

## Data Availability

The Unified Human Gastrointestinal Protein (UHGP) catalogue is available from the MGnify FTP site (http://ftp.ebi.ac.uk/pub/databases/metagenomics/mgnify_genomes/) alongside other data provided by original authors^16^. Metagenomic samples used for validation are deposited in the EMBL-EBI European Nucleotide Archive (ENA) under accession numbers PRJEB37249 and PRJNA762199. Full UniProtKB/Swiss-Prot 2019_02 release is available from UnitProt FTP (https://ftp.uniprot.org/pub/databases/uniprot/previous_major_releases/release-2019_02/). Preprocessed data (Bacterial SwissProt) can be downloaded using a script available in our code repository (https://github.com/ardigen/microbiome-protein-embeddings).

## Code availability

Code used in the analyses is available at https://github.com/ardigen/microbiome-protein-embeddings.

## References

[1] Turnbaugh PJ (2009). A core gut microbiome in obese and lean twins. Nature.

[2] Gevers D (2014). The treatment-naive microbiome in new-onset Crohn’s disease. Cell Host Microbe.

[3] Lloyd-Price J (2019). Multi-omics of the gut microbial ecosystem in inflammatory bowel diseases. Nature.

[4] Franzosa EA (2019). Gut microbiome structure and metabolic activity in inflammatory bowel disease. Nat Microbiol.

[5] Vatanen T (2019). Genomic variation and strain-specific functional adaptation in the human gut microbiome during early life. Nat Microbiol.

[6] Vatanen T (2018). The human gut microbiome in early-onset type 1 diabetes from the TEDDY study. Nature.

[7] Helmink BA, Khan MAW, Hermann A, Gopalakrishnan V, Wargo JA (2019). The microbiome, cancer, and cancer therapy. Nat. Med..

[8] Sepich-Poore GD (2021). The microbiome and human cancer. Science.

[9] Valles-Colomer M (2019). The neuroactive potential of the human gut microbiota in quality of life and depression. Nat Microbiol.

[10] Nguyen TT, Hathaway H, Kosciolek T, Knight R, Jeste DV (2019). Gut microbiome in serious mental illnesses: A systematic review and critical evaluation. Schizophr. Res..

[11] Cryan JF, Dinan TG (2012). Mind-altering microorganisms: The impact of the gut microbiota on brain and behaviour. Nat. Rev. Neurosci..

[12] Jo J-H, Kennedy EA, Kong HH (2016). Research techniques made simple: Bacterial 16S ribosomal RNA gene sequencing in cutaneous research. J. Invest. Dermatol..

[13] Altschul SF, Gish W, Miller W, Myers EW, Lipman DJ (1990). Basic local alignment search tool. J. Mol. Biol..

[14] Eddy SR (1995). Multiple alignment using hidden Markov models. Proc. Int. Conf. Intell. Syst. Mol. Biol..

[15] Prakash T, Taylor TD (2012). Functional assignment of metagenomic data: Challenges and applications. Brief. Bioinform..

[16] Almeida A (2020). A unified catalog of 204,938 reference genomes from the human gut microbiome. Nat. Biotechnol..

[17] Zhou N (2019). The CAFA challenge reports improved protein function prediction and new functional annotations for hundreds of genes through experimental screens. Genome Biol..

[18] LeCun Y, Bengio Y, Hinton G (2015). Deep learning. Nature.

[19] Angermueller C, Pärnamaa T, Parts L, Stegle O (2016). Deep learning for computational biology. Mol. Syst. Biol..

[20] Hoarfrost A, Aptekmann A, Farfañuk G, Bromberg Y (2020). Shedding light on microbial dark matter with a universal language of Life. bioRxiv.

[21] Senior AW (2020). Improved protein structure prediction using potentials from deep learning. Nature.

[22] Senior AW (2019). Protein structure prediction using multiple deep neural networks in the 13th Critical Assessment of Protein Structure Prediction (CASP13). Proteins.

[23] Jumper J (2021). Highly accurate protein structure prediction with AlphaFold. Nature.

[24] Kryshtafovych A, Schwede T, Topf M, Fidelis K, Moult J (2019). Critical assessment of methods of protein structure prediction (CASP)-Round XIII. Proteins.

[25] Kryshtafovych A, Schwede T, Topf M, Fidelis K, Moult J (2021). Critical assessment of methods of protein structure prediction (CASP)-Round XIV. Proteins.

[26] Ashburner M (2000). Gene ontology: Tool for the unification of biology The Gene Ontology Consortium. Nat. Genet..

[27] Bairoch A (2000). The ENZYME database in 2000. Nucleic Acids Res..

[28] Li Y (2018). DEEPre: Sequence-based enzyme EC number prediction by deep learning. Bioinformatics.

[29] Zou Z, Tian S, Gao X, Li Y (2018). mlDEEPre: Multi-functional enzyme function prediction with hierarchical multi-label deep learning. Front. Genet..

[30] Kulmanov M, Khan MA, Hoehndorf R, Wren J (2018). DeepGO: Predicting protein functions from sequence and interactions using a deep ontology-aware classifier. Bioinformatics.

[31] Kulmanov M, Hoehndorf R (2020). DeepGOPlus: Improved protein function prediction from sequence. Bioinformatics.

[32] Duong D (2020). Annotating gene ontology terms for protein sequences with the transformer model. bioRxiv.

[33] Nauman M, Ur Rehman H, Politano G, Benso A (2018). Beyond homology transfer: Deep learning for automated annotation of proteins. Int. J. Grid Util. Comput..

[34] Sureyya Rifaioglu A, Doğan T, Jesus Martin M, Cetin-Atalay R, Atalay V (2019). DEEPred: Automated protein function prediction with multi-task feed-forward deep neural networks. Sci. Rep..

[35] Saiful Islam, S. M. & Hasan, M. M. DEEPGONET: Multi-label prediction of GO annotation for protein from sequence using cascaded convolutional and recurrent network. In *2018 21st International Conference of Computer and Information Technology (ICCIT)* 1–6. 10.1109/ICCITECHN.2018.8631921 (2018).

[36] Seo S, Oh M, Park Y, Kim S (2018). DeepFam: Deep learning based alignment-free method for protein family modeling and prediction. Bioinformatics.

[37] Bileschi ML (2019). Using deep learning to annotate the protein universe. bioRxiv.

[38] Schwartz AS (2018). Deep semantic protein representation for annotation, discovery, and engineering. bioRxiv.

[39] Rao R, Wallach H (2019). Evaluating protein transfer learning with TAPE. Advances in Neural Information Processing Systems.

[40] Rives A (2021). Biological structure and function emerge from scaling unsupervised learning to 250 million protein sequences. Proc. Natl. Acad. Sci. U. S. A..

[41] Littmann M, Heinzinger M, Dallago C, Olenyi T, Rost B (2021). Embeddings from deep learning transfer GO annotations beyond homology. Sci. Rep..

[42] Villegas-Morcillo A (2021). Unsupervised protein embeddings outperform hand-crafted sequence and structure features at predicting molecular function. Bioinformatics.

[43] Staerk H, Dallago C, Heinzinger M, Rost B (2021). Light attention predicts protein location from the language of life. bioRxiv.

[44] Elnaggar, A. *et al.* ProtTrans: Towards cracking the language of lifes code through self-supervised deep learning and high performance computing. *IEEE Trans. Pattern Anal. Mach. Intell.* (2021).

[45] Alley EC, Khimulya G, Biswas S, AlQuraishi M, Church GM (2019). Unified rational protein engineering with sequence-based deep representation learning. Nat. Methods.

[46] Madani A (2020). ProGen: Language modeling for protein generation. bioRxiv.

[47] Thireou T, Reczko M (2007). Bidirectional Long Short-Term Memory Networks for predicting the subcellular localization of eukaryotic proteins. IEEE/ACM Trans. Comput. Biol. Bioinform..

[48] McInnes, L., Healy, J. & Melville, J. UMAP: Uniform Manifold Approximation and Projection for Dimension Reduction. *arXiv [stat.ML]* (2018).

[49] Steinegger M, Söding J (2018). Clustering huge protein sequence sets in linear time. Nat. Commun..

[50] Mistry J (2021). Pfam: The protein families database in 2021. Nucleic Acids Res..

[51] Baeza-Yates R, Ribeiro-Neto B (1999). Modern Information Retrieval.

[52] Manning C, Raghavan P, Schütze H (2010). Introduction to information retrieval. Nat. Lang. Eng..

[53] Zhang M-L, Zhou Z-H (2014). A review on multi-label learning algorithms. IEEE Trans. Knowl. Data Eng..

[54] Rao R, Meier J, Sercu T, Ovchinnikov S, Rives A (2020). Transformer protein language models are unsupervised structure learners. bioRxiv.

[55] Gligorijević V (2021). Structure-based protein function prediction using graph convolutional networks. Nat. Commun..

[56] Perdigão N (2015). Unexpected features of the dark proteome. Proc. Natl. Acad. Sci. U. S. A..

[57] Miravet-Verde S (2019). Unraveling the hidden universe of small proteins in bacterial genomes. Mol. Syst. Biol..

[58] Sberro H (2019). Large-scale analyses of human microbiomes reveal thousands of small, novel genes. Cell.

[59] Koehler Leman J, Mueller BK, Gray JJ (2017). Expanding the toolkit for membrane protein modeling in Rosetta. Bioinformatics.

[60] Heinzinger M (2019). Modeling aspects of the language of life through transfer-learning protein sequences. BMC Bioinform..

[61] Parada Venegas D (2019). Short chain fatty acids (SCFAs)-mediated gut epithelial and immune regulation and its relevance for inflammatory bowel diseases. Front. Immunol..

[62] Xiao S, Jiang S, Qian D, Duan J (2020). Modulation of microbially derived short-chain fatty acids on intestinal homeostasis, metabolism, and neuropsychiatric disorder. Appl. Microbiol. Biotechnol..

[63] Alexander C, Swanson KS, Fahey GC, Garleb KA (2019). Perspective: Physiologic importance of short-chain fatty acids from nondigestible carbohydrate fermentation. Adv. Nutr..

[64] Palacios S, Starai VJ, Escalante-Semerena JC (2003). Propionyl coenzyme A is a common intermediate in the 1,2-propanediol and propionate catabolic pathways needed for expression of the prpBCDE operon during growth of Salmonella enterica on 1,2-propanediol. J. Bacteriol..

[65] Sievers F, Higgins DG (2018). Clustal Omega for making accurate alignments of many protein sequences. Protein Sci..

[66] Radivojac P (2013). A large-scale evaluation of computational protein function prediction. Nat. Methods.

[67] Eddy SR (2009). A new generation of homology search tools based on probabilistic inference. Genome Inform..

[68] Guo Y, Wang B, Li W, Yang B (2018). Protein secondary structure prediction improved by recurrent neural networks integrated with two-dimensional convolutional neural networks. J. Bioinform. Comput. Biol..

[69] Xuan W, Liu N, Huang N, Li Y, Wang J (2020). CLPred: A sequence-based protein crystallization predictor using BLSTM neural network. Bioinformatics.

[70] Rao RM (2021). MSA transformer. Int. Conf. Mach. Learn..

[71] Paszke A (2019). PyTorch: An imperative style, high-performance deep learning library. Adv. Neural Inf. Process. Syst..

[72] Molinaro A (2020). Imidazole propionate is increased in diabetes and associated with dietary patterns and altered microbial ecology. Nat. Commun..

[73] Li D, Liu CM, Luo R, Sadakane K, Lam TW (2015). MEGAHIT: An ultra-fast single-node solution for large and complex metagenomics assembly via succinct de Bruijn graph. Bioinformatics.

[74] Hyatt D (2010). Prodigal: Prokaryotic gene recognition and translation initiation site identification. BMC Bioinform..

[75] El-Gebali S (2019). The Pfam protein families database in 2019. Nucleic Acids Res..

[76] Steinegger M, Söding J (2017). MMseqs2 enables sensitive protein sequence searching for the analysis of massive data sets. Nat. Biotechnol..

[77] Ghandi M, Lee D, Mohammad-Noori M, Beer MA (2014). Enhanced regulatory sequence prediction using gapped k-mer features. PLoS Comput. Biol..

[78] Pedregosa F (2011). Scikit-learn: Machine learning in Python. J. Mach. Learn. Res..

[79] Becht E (2018). Dimensionality reduction for visualizing single-cell data using UMAP. Nat. Biotechnol..

[80] Sievers F (2011). Fast, scalable generation of high-quality protein multiple sequence alignments using Clustal Omega. Mol. Syst. Biol..

[81] Cock PJA (2009). Biopython: Freely available Python tools for computational molecular biology and bioinformatics. Bioinformatics.

[82] Waterhouse AM, Procter JB, Martin DMA, Clamp M, Barton GJ (2009). Jalview Version 2: A multiple sequence alignment editor and analysis workbench. Bioinformatics.

