# Supplementary material for: Deep embeddings to comprehend and visualize microbiome protein space
Source: Sci Rep. 2022 Jun 20;12:10332. doi: 10.1038/s41598-022-14055-7 (PMC9209496; doi:10.1038/s41598-022-14055-7)

Supplementary data 2. Domain sequence alignment between two outlying proteins (BUK_OCEIH and BUK_DEIRA) and PF00696 domain sequences from other phosphotransferases (EC 2.7.2.8, 2.7.2.2, 2.7.2.4 and 2.7.2.11)

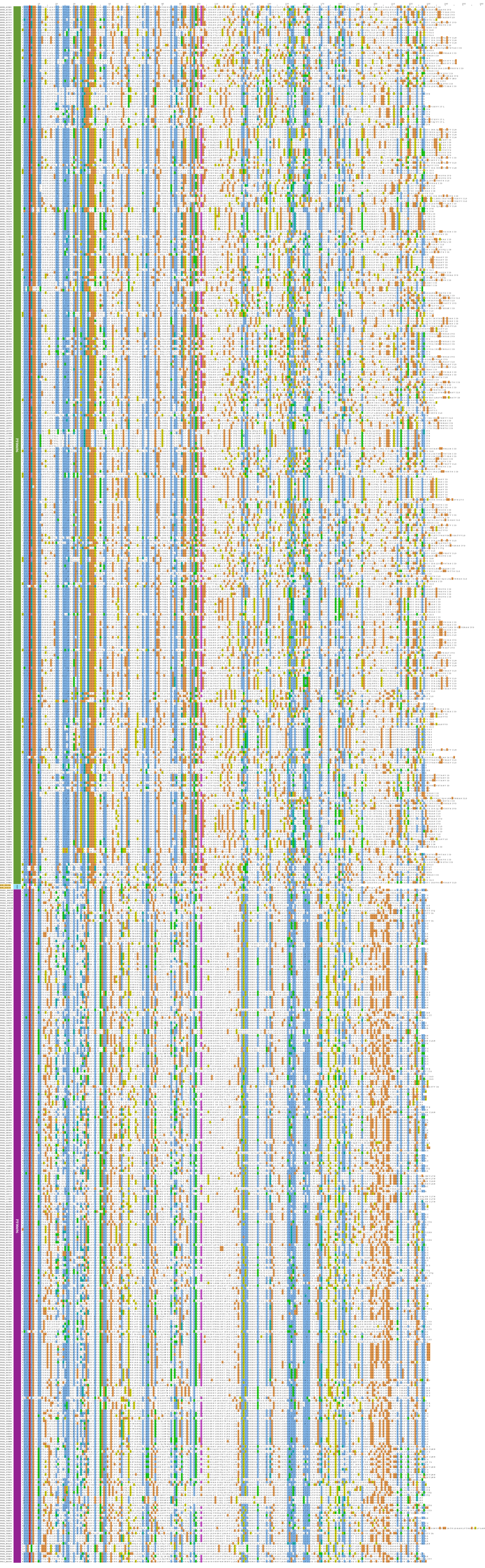

Supplement: Supplementary file 2 — Supplementary Information 2. [file 41598_2022_14055_MOESM2_ESM.pdf]
