# Supplementary material for: Deep embeddings to comprehend and visualize microbiome protein space
Source: Sci Rep. 2022 Jun 20;12:10332. doi: 10.1038/s41598-022-14055-7 (PMC9209496; doi:10.1038/s41598-022-14055-7)

Supplementary data 3. Domain sequence alignment between two outlying proteins (BUK_OCEIH and BUK_DEIRA) and PF00871 domain sequences from other phosphotransferases (EC 2.7.2.1, 2.7.2.15 and 2.7.2.7)

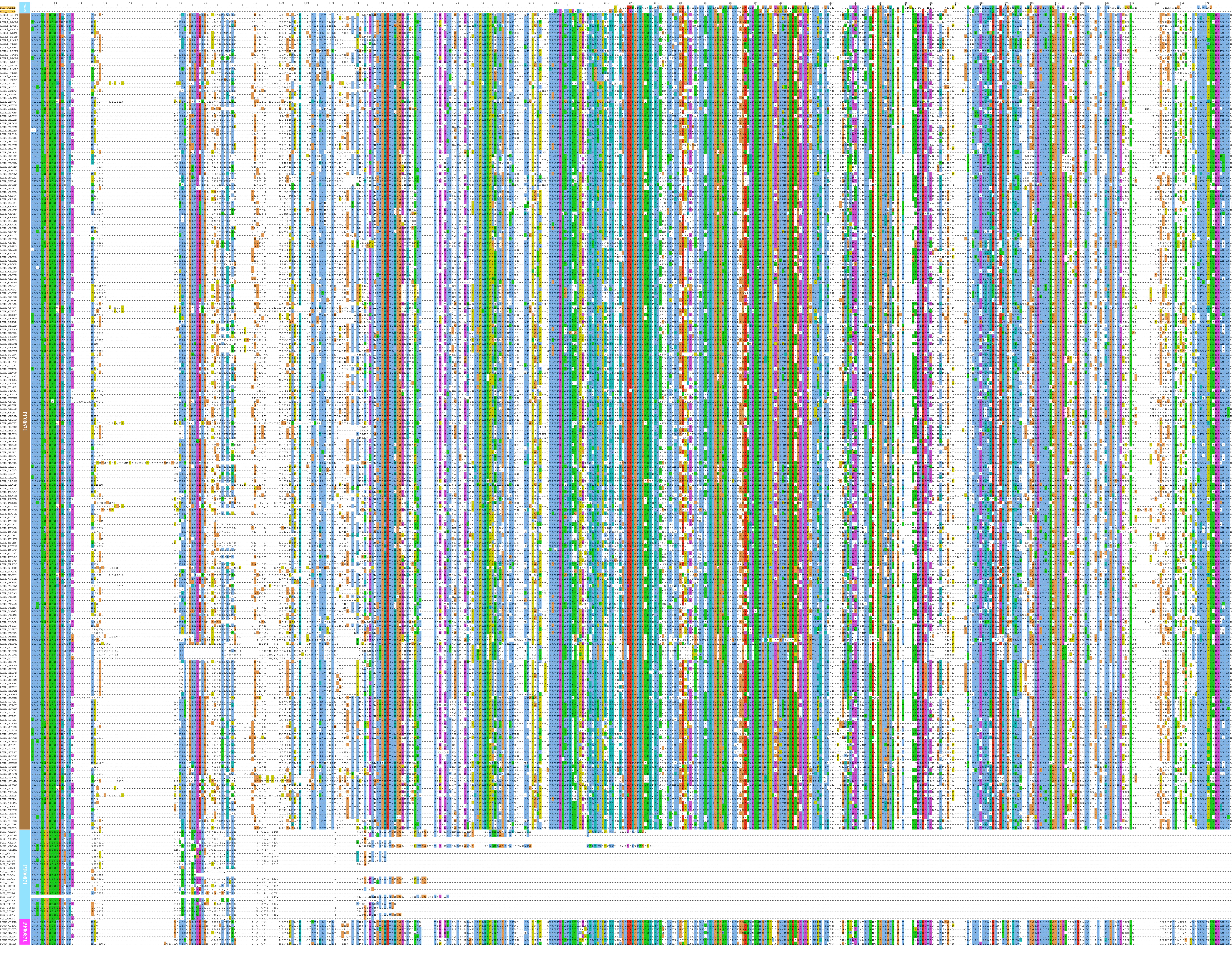

Supplement: Supplementary file 3 — Supplementary Information 3. [file 41598_2022_14055_MOESM3_ESM.pdf]
