# Supplementary material for: Deep embeddings to comprehend and visualize microbiome protein space
Source: Sci Rep. 2022 Jun 20;12:10332. doi: 10.1038/s41598-022-14055-7 (PMC9209496; doi:10.1038/s41598-022-14055-7)
Supplement: Supplementary file 4 — Supplementary Information 4. [file 41598_2022_14055_MOESM4_ESM.pdf]

Supplementary information

Supplementary Table 1. Summary of samples selected from PRJEB37249 project for metagenomic validation of the deep embedding model.

		ECE	
SampleId (Run Accession)	n. of proteins	UHGP model	Pfam model
ERR4086424	38,410	11.421	15.400
ERR4086425	55,508	10.932	15.173
ERR4086426	41,077	10.842	15.176
ERR4086427	51,965	10.813	15.131
ERR4086428	61,933	10.810	15.123
ERR4086429	59,977	10.779	15.103
ERR4086430	108,120	10.204	17.066
ERR4086431	23,021	11.326	15.168
ERR4086432	22,640	10.575	14.860
ERR4086433	37,703	11.528	15.201
mean	50,035	10.923	15.340
std	24,711	0.400	0.620

Supplementary Table 2. Summary of samples selected from PRJNA762199 project for metagenomic validation of the deep embedding model.

		ECE	
SampleId (Run Accession)	n. of proteins	UHGP model	Pfam model
SRR16681825	564,892	8.403	13.435
SRR16681826	488,248	8.098	13.252
SRR16681827	612,190	9.517	13.831
SRR16681828	587,146	8.320	13.397
SRR16681829	334,698	8.234	13.376
SRR16681831	593,731	8.259	13.391
SRR16681832	471,654	8.502	13.479
SRR16681833	467,390	8.522	13.364
SRR16681834	566,267	8.512	13.481
SRR16681835	429,537	8.471	13.393
mean	511,575	8.484	13.440
std	88,521	0.389	0.152

Supplementary Table 3. The degree of correctness in the recovery of labels using deep, k-mer-based, or amino acid frequency representations, and MMseqs2 - state-of-the-art proteins search tool. The measure of the recovery is Intersection over Union (IoU) between original labels and a set of labels from the nearest neighbors.

Label	Representation	Neighborhood size					
		1	3	15	51	101	201
SUPFAM	3-mers TF-IDF	0.836	0.763	0.489	0.286	0.199	0.131
	AA freq.	0.695	0.582	0.266	0.107	0.061	0.033
	Deep Embeddings	0.956	0.936	0.848	0.731	0.627	0.455
	MMseqs2	0.988	0.979	0.932	0.831	0.734	0.571
Gene3D	3-mers TF-IDF	0.848	0.778	0.503	0.291	0.202	0.136
	AA freq.	0.716	0.607	0.288	0.114	0.064	0.033
	Deep Embeddings	0.959	0.940	0.856	0.739	0.633	0.453
	MMseqs2	0.990	0.981	0.939	0.840	0.754	0.593
Pfam	3-mers TF-IDF	0.804	0.725	0.441	0.245	0.168	0.108
	AA freq.	0.663	0.545	0.233	0.086	0.047	0.022
	Deep Embeddings	0.927	0.898	0.781	0.641	0.525	0.356
	MMseqs2	0.973	0.956	0.888	0.772	0.669	0.496
KO	3-mers TF-IDF	0.833	0.756	0.467	0.260	0.177	0.112
	AA freq.	0.684	0.572	0.250	0.092	0.049	0.023
	Deep Embeddings	0.939	0.912	0.800	0.653	0.529	0.350
	MMseqs2	0.977	0.962	0.910	0.811	0.711	0.525
InterPro	3-mers TF-IDF	0.793	0.713	0.431	0.238	0.162	0.104
	AA freq.	0.655	0.535	0.228	0.085	0.047	0.023
	Deep Embeddings	0.914	0.882	0.756	0.611	0.496	0.334
	MMseqs2	0.962	0.939	0.860	0.736	0.631	0.461
EC number	3-mers TF-IDF	0.815	0.730	0.415	0.195	0.115	0.062
	AA freq.	0.649	0.527	0.199	0.061	0.030	0.016
	Deep Embeddings	0.932	0.904	0.788	0.627	0.486	0.296
	MMseqs2	0.970	0.956	0.897	0.791	0.675	0.463
GO	3-mers TF-IDF	0.793	0.715	0.428	0.225	0.148	0.090
	AA freq.	0.667	0.559	0.259	0.11	0.066	0.038
	Deep Embeddings	0.896	0.857	0.702	0.523	0.412	0.280
	MMseqs2	0.929	0.898	0.782	0.627	0.524	0.378
eggNOG	3-mers TF-IDF	0.777	0.699	0.420	0.227	0.152	0.093
	AA freq.	0.624	0.516	0.220	0.078	0.041	0.019
	Deep Embeddings	0.890	0.857	0.720	0.557	0.435	0.277
	MMseqs2	0.941	0.923	0.841	0.704	0.576	0.383
Phylum	3-mers TF-IDF	0.833	0.760	0.457	0.224	0.144	0.102
	AA freq.	0.776	0.698	0.403	0.210	0.151	0.114
	Deep Embeddings	0.847	0.779	0.505	0.252	0.154	0.099
	MMseqs2	0.924	0.881	0.693	0.405	0.210	0.089
Order	3-mers TF-IDF	0.649	0.520	0.149	0.055	0.037	0.026
	AA freq.	0.566	0.436	0.133	0.057	0.040	0.029
	Deep Embeddings	0.635	0.505	0.161	0.059	0.036	0.024
	MMseqs2	0.769	0.663	0.260	0.068	0.034	0.020
Family	3-mers TF-IDF	0.565	0.417	0.094	0.035	0.023	0.016
	AA freq.	0.490	0.349	0.091	0.038	0.025	0.018
	Deep Embeddings	0.539	0.392	0.098	0.035	0.021	0.013
	MMseqs2	0.661	0.516	0.122	0.035	0.018	0.011
Genus	3-mers TF-IDF	0.411	0.275	0.060	0.023	0.015	0.010
	AA freq.	0.366	0.241	0.061	0.025	0.017	0.012
	Deep Embeddings	0.390	0.260	0.060	0.021	0.012	0.008
	MMseqs2	0.464	0.32	0.066	0.019	0.010	0.006

Supplementary Table 4. Annotation of proteins that are clustered in UMAP visualization to functional databases such as KEGG, GO, Pfam and annotation to EC number. We can see that proteins within a cluster share annotations.

PROTEIN NAME	KO	EC number	Pfam
**Fig 4A - proteins transferring alkyl or aryl groups, other than methyl groups**			
UDP-N-acetylglucosamine 1-carboxyvinyltransferase	*K00790*	EC: 2.5.1.7	**PF00275**, EPSP_synthase
3-phosphoshikimate 1-carboxyvinyltransferase	*K00800*	EC: 2.5.1.19	
**Fig 4B - GTP binding proteins**			
Elongation factor G	*K02355*	-	**PF00679**, EFG_C<br>**PF03764**, EFG_IV<br>**PF00009**, GTP_EFTU<br>**PF03144**, GTP_EFTU_D2
Peptide chain release factor 3	*K02837*	-	**PF00009,** GTP_EFTU<br>(**PF03144**), GTP_EFTU_D2<br>**PF16658**, RF3_C
**Fig 4E - ribosomal proteins**			
30S ribosomal protein S1	*K02961*	-	**PF00575**, S1
50S ribosomal protein L14	*K02874*		**PF00238**, Ribosomal_L14
50S ribosomal protein L36	*K02919*	-	**PF00444**, Ribosomal_L36
50S ribosomal protein L35	*K02916*		**PF01632**, Ribosomal_L35p
50S ribosomal protein L15	*K02876*		**PF00828**, Ribosomal_L27A
**Fig 4D - tRNA ligases**			
Cysteine--tRNA ligase	*K01883*	EC: 6.1.1.16	**PF09190**, DALR_2<br>**PF01406**, tRNA-synt_1e
Arginine--tRNA ligase	*K01887*	EC: 6.1.1.19	**PF03485**, Arg_tRNA_synt_N<br>**PF05746**, DALR_1<br>**PF00750**, tRNA-synt_1d
Glutamate--tRNA ligase	*K01885*	EC: 6.1.1.17	**PF00749**, tRNA-synt_1c
Glutamine--tRNA ligase	*K01886*	EC: 6.1.1.18	**PF00749**, tRNA-synt_1c<br>**PF03950**, tRNA-synt_1c_C
Glycine--tRNA ligase	*K01880*	EC: 6.1.1.14	**PF03129**, HGTP_anticodon<br>**PF00587**, tRNA-synt_2b
Valine---tRNA ligase	*K01873*	EC: 6.1.1.9	**PF08264**, Anticodon_1<br>**PF00133**, tRNA-synt_1<br>**PF10458**, Val_tRNA-synt_C
isoleucyl-tRNA synthetase	*K01870*	*EC: 6.1.1.5*	**PF08264**, Anticodon_1<br>**PF00133**, tRNA-synt_1<br>**(PF06827),** zf-FPG_IleRS

Supplementary Table 5. Description (i.e.: EC number, name, domain and number of proteins in Bacterial SwissProt) of the chosen EC 2.7.2 family that was used for a real life use case.

EC number	Name	Pfam domain architecture	Number of proteins
2.7.2.1	Acetate kinase	PF00871	226
2.7.2.2	Carbamate kinase	PF00871	18
2.7.2.3	Phosphoglycerate kinase	PF00162<br>PF00162||PF00121	377<br>1
2.7.2.4	Aspartate kinase	PF00696<br>PF00696||PF01842||PF13840<br>PF00696||PF13840<br>PF00696||PF01842<br>PF00696||PF00742||PF03447<br>PF00696||PF01842||PF13840||PF00742||PF03447<br>PF00696||PF13840||PF00742||PF03447	8<br>13<br>4<br>1<br>4<br>2<br>1
2.7.2.5	Transferred entry: 6.3.4.16	-	0
2.7.2.6	Formate kinase	-	0
2.7.2.7	Butyrate kinase	PF00871	27
2.7.2.8	Acetylglutamate kinase	PF00696<br>PF00696||PF04768	344<br>3
2.7.2.9	Transferred entry: 6.3.5.5	-	0
2.7.2.10	Phosphoglycerate kinase (GTP)	-	0
2.7.2.11	Glutamate 5-kinase	PF00696<br>PF00696||PF01472	34<br>230
2.7.2.12	Acetate kinase (diphosphate)	-	0
2.7.2.13	Deleted entry	-	0
2.7.2.14	Branched-chain-fatty-acid kinase	-	0
2.7.2.15	Propionate kinase	PF00871	14
2.7.2.16	2-phosphoglycerate kinase	-	0
2.7.2.17	[Amino-group carrier protein]-L-2-aminoadipate 6-kinase	-	0

Supplementary Table 6. Efficiency summary of operations used in the article. Time of each operation was measured 10 times for the 1,000 proteins column and 3 times for the 10,000 proteins column. Mean and standard deviation was then calculated. 4 CPUs were used in most cases with an exception of operations marked with [GPU], which were processed on a Tesla V100 GPU. **Search proteins** - we search for the nearest proteins of random 1,000 or 10,000 proteins from Bacterial SwissProt in the remaining proteins from Bacterial SwissProt. **Distance matrix -** calculating full distance matrix of random 1,000 or 10,000 proteins from Bacterial SwissProt.

	Name	Time for 1,000 proteins (N=10)	Time for 10,000 proteins (N=3)
Individual operations	Embed proteins	6 min 29 s ± 1.8 s	1h 5min 48s ± 29.2 s
	Embed proteins [GPU]	25.8 s ± 1.1 s	2 min 53 s ± 0.5 s
	Search proteins - Embeddings [k=3]	8.53 s ± 0.3 s	9.2 s ± 0.6 s
	Search proteins - Embeddings [k=201]	28.7 s ± 3.3 s	31.3 s ± 2.0 s
	Search proteins - MMseqs2	17.5 s ± 1.2 s	1min 44s ± 1.47 s
	Search proteins - MMseqs2 [-e inf -s 9.0]	1 min 10 s ± 9.7 s	5min 36s ± 5.67 s
	Distance matrix - Embeddings	132 ms ± 8.6 ms	655 ms ± 81.9 ms
	Distance matrix - ClustalO	1 min 58.2 s ± 6.1 s	3h 23 min
Example use cases	Embed & Search proteins [CPU, k=3]	6 min 37s ± 2.1 s	1h 5min 57s ± 29.8 s
	Embed & Search proteins [GPU, k=3]	34.3 s ± 1.4 s	3 min 2 s ± 1.1 s
	Search proteins - MMseqs2	**17.5 s ± 1.2 s**	**1min 44s ± 1.47 s**
	Embed & Search proteins [CPU, k=201]	6 min 58 s ± 5.1 s	1h 6min 18s ± 31 s
	Embed & Search proteins [GPU, k=201]	**54.5 s ± 5.4 s**	**3 min 24 s ± 2.0 s**
	Search proteins - MMseqs2 [-e inf -s 9.0]	1 min 10 s ± 9.69 s	5min 36s ± 5.67 s
	Embed & Distance matrix [CPU]	6 min 29 s ± 1.85 s	1h 5min 48s ± 29.2 s
	Embed & Distance matrix [GPU]	**25.9 s ± 1.2 s**	**2 min 53 s ± 0.5 s**
	Distance matrix - ClustalO	1 min 58.2 s ± 6.1 s	3h 12 min ± 6 min

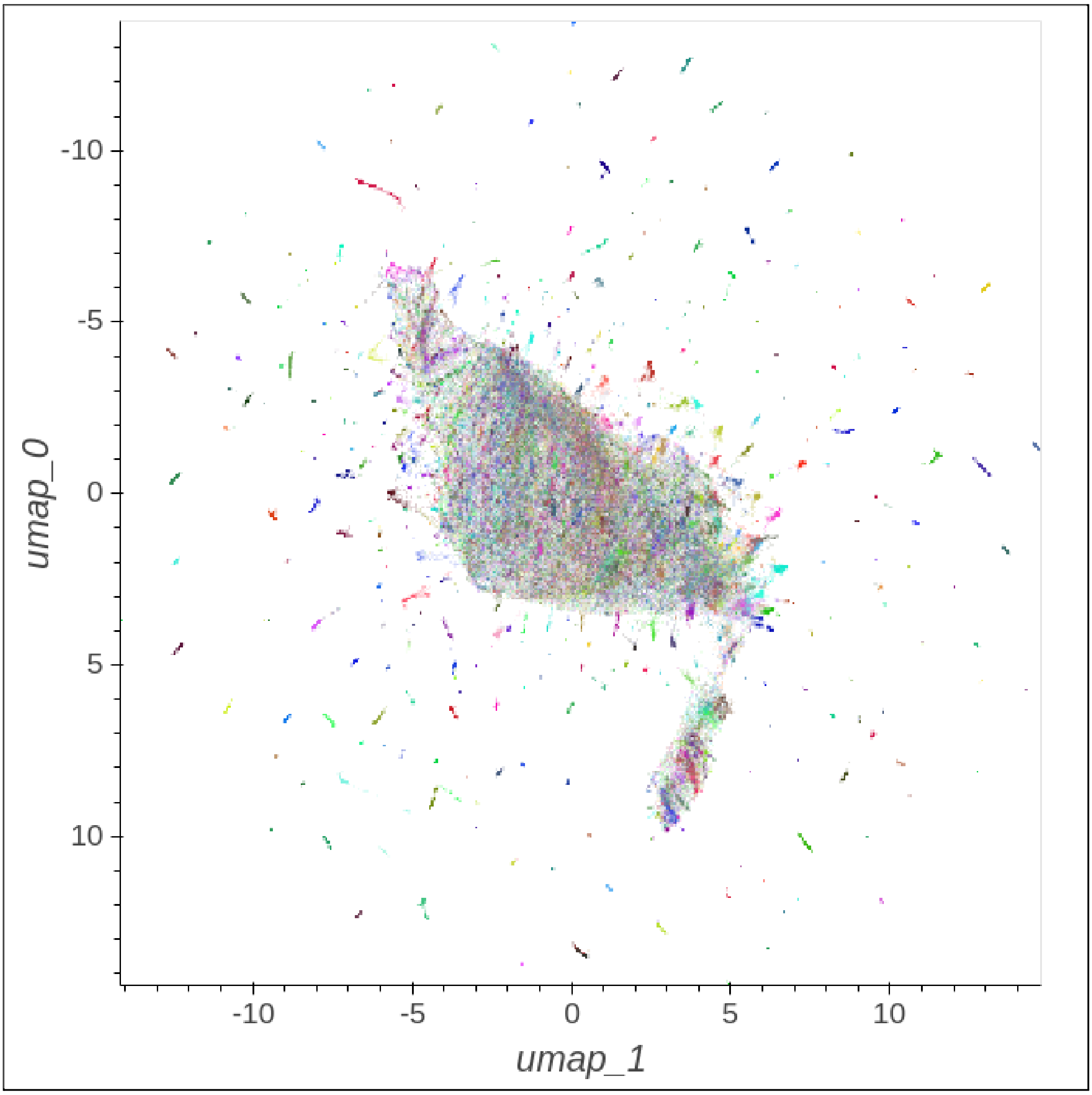

Supplementary Figure 1. UMAP visualization of the k-mer protein representations space colored according to Kegg Orthology ID (KO). We see separate groups of proteins, however, most of them are mixed up in the middle.

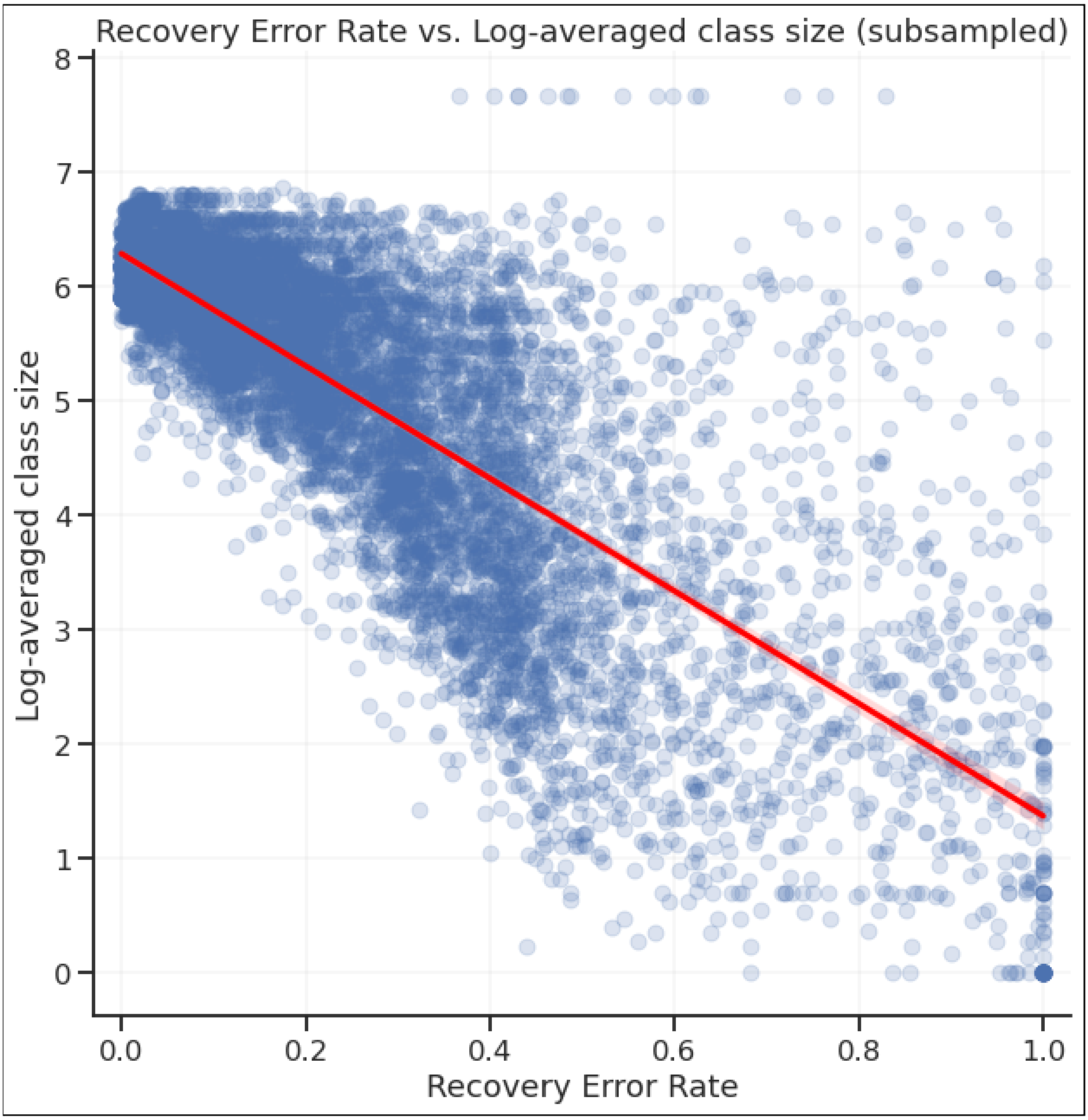

Supplementary Figure 2. Relationship between Recovery Error Rate and the size of the class to which the protein belongs. Red lines shows the the correlation (r=-0.776, N=200,115)

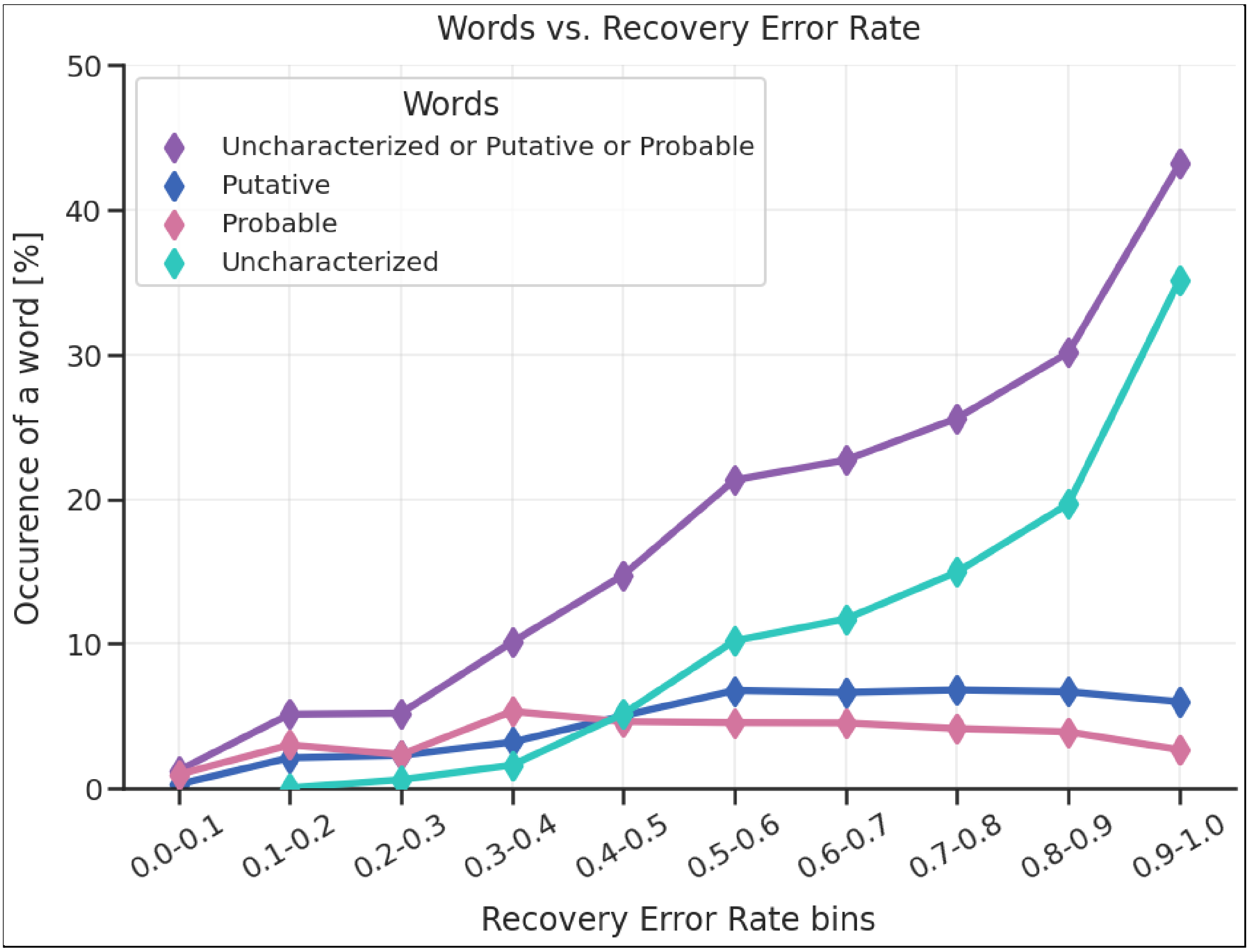

Supplementary Figure 3. The relationship between Recovery Error Rate and the occurrence of the words "Uncharacterized", "Putative", or "Probable"

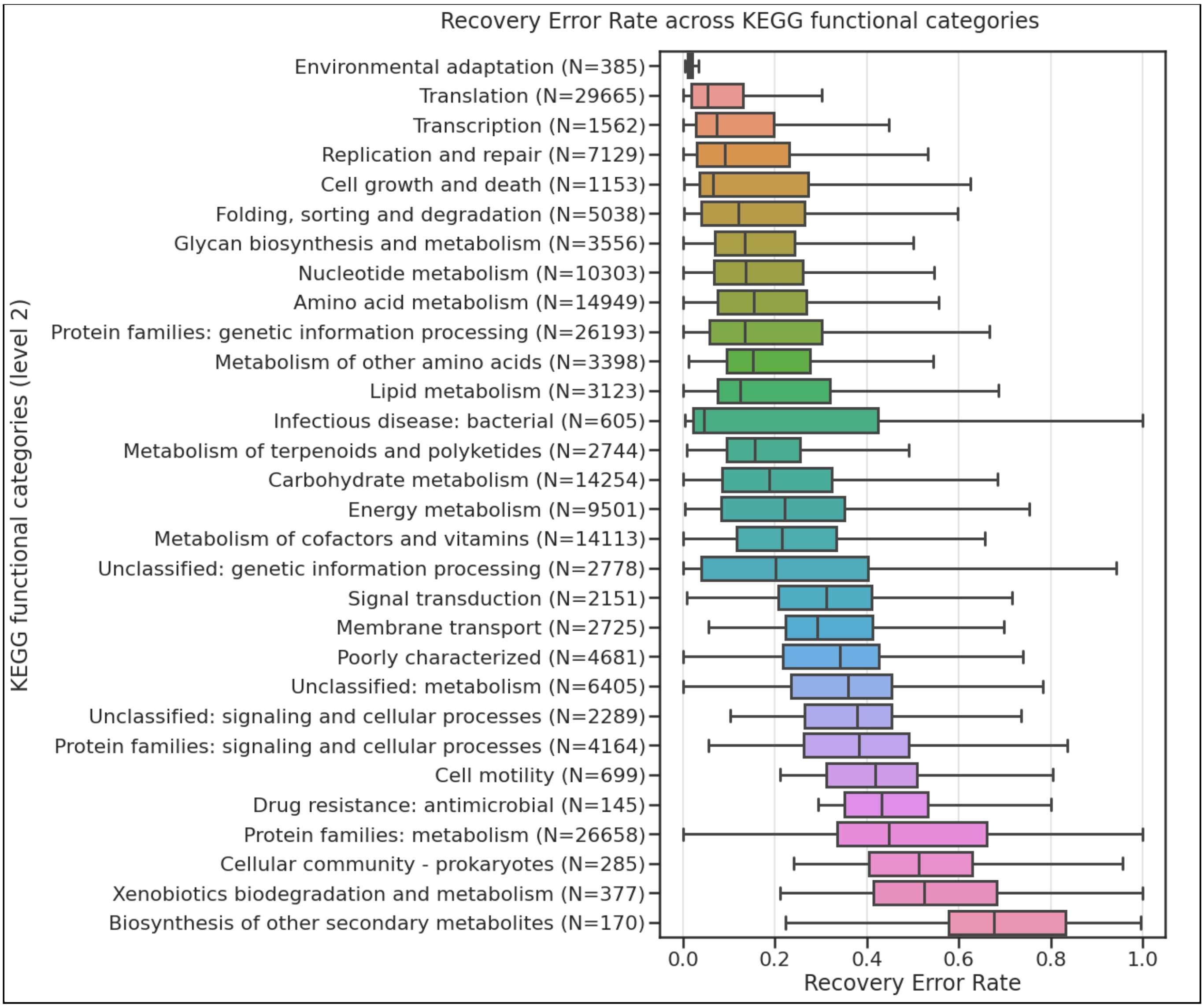

Supplementary Figure 4. Distribution of Recovery Error Rate across KEGG functional categories. Categories are sorted by their mean Recovery Error Rate. Only functional categories with N > 100 are shown.

Supplementary Table 7. References to the databases used in our analyses.

Database	Link to database
**SUPFAM**[1]	https://supfam.org
**GENE 3D**[2]	http://gene3d.biochem.ucl.ac.uk
**InterPro**[3]	https://www.ebi.ac.uk/interpro/
**KO (KEGG Orthology)**[4]	https://www.kegg.jp
**GO (Gene Ontology)**[5]	http://geneontology.org
**eggNOG**[6]	http://eggnog5.embl.de/#/app/home
**EC number**[7]	https://enzyme.expasy.org
**Pfam**[8]	http://pfam.xfam.org
**Taxonomy**[9]	https://www.ncbi.nlm.nih.gov/taxonomy

References

1. Pandurangan, A. P., Stahlhacke, J., Oates, M. E., Smithers, B. & Gough, J. The SUPERFAMILY 2.0 database: a significant proteome update and a new webserver. *Nucleic Acids Res.* **47**, D490–D494 (2019).
2. Lees, J. *et al.* Gene3D: a domain-based resource for comparative genomics, functional annotation and protein network analysis. *Nucleic Acids Res.* **40**, D465–71 (2012).
3. Blum, M. *et al.* The InterPro protein families and domains database: 20 years on. *Nucleic Acids Res.* **49**, D344–D354 (2021).
4. Kanehisa, M., Furumichi, M., Sato, Y., Ishiguro-Watanabe, M. & Tanabe, M. KEGG: integrating viruses and cellular organisms. *Nucleic Acids Research* vol. 49 D545–D551 (2021).
5. The Gene Ontology resource: enriching a GOld mine. *Nucleic Acids Res.* **49**, D325–D334 (2021).
6. Huerta-Cepas, J. *et al.* eggNOG 5.0: a hierarchical, functionally and phylogenetically annotated orthology resource based on 5090 organisms and 2502 viruses. *Nucleic Acids Res.* **47**, D309–D314 (2019).
7. Duvaud, S. *et al.* Expasy, the Swiss Bioinformatics Resource Portal, as designed by its users. *Nucleic Acids Res.* **49**, W216–W227 (2021).
8. Mistry, J. *et al.* Pfam: The protein families database in 2021. *Nucleic Acids Res.* **49**, D412–D419 (2021).
9. Schoch, C. L. *et al.* NCBI Taxonomy: a comprehensive update on curation, resources and tools. *Database* **2020**, (2020).
