# Supplementary material for: Deep embeddings to comprehend and visualize microbiome protein space
Source: Sci Rep. 2022 Jun 20;12:10332. doi: 10.1038/s41598-022-14055-7 (PMC9209496; doi:10.1038/s41598-022-14055-7)

Supplementary Data 1. Domain sequence alignment between two outlying proteins (BUK_OCEIH and BUK_DEIRA) and other butyrate kinase proteins (EC 2.7.2.7)

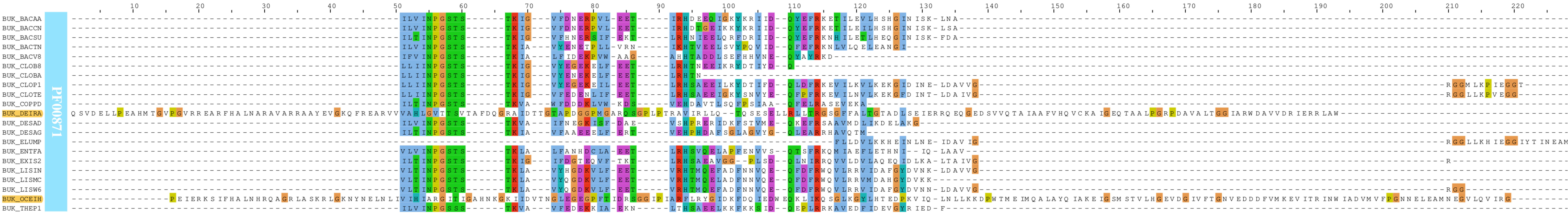

Supplement: Supplementary file 1 — Supplementary Information 1. [file 41598_2022_14055_MOESM1_ESM.pdf]
